# Conflict resolution of the beams: CT vs. MRI in recurrent hernia detection: a systematic review and meta-analysis of mesh visualization and other outcomes

**DOI:** 10.1007/s10029-025-03308-9

**Published:** 2025-03-28

**Authors:** Ahmed Abdelsamad, Ibrahim Khalil, Mohammed Khaled Mohammed, Aya sayed ahmed said Serour, Zeyad M. Wesh, Omar Zaree, Mohamed Abdelmohsen Bedewi, Zainab Hussein, Torsten Herzog, Khaled Ashraf Mohamed, Florian Gebauer

**Affiliations:** 1 Department of Surgery II, University of Witten/Herdecke, 58455 Witten, Germany; 2 Oncological Surgery Department, Section Head of Robotic Surgery, Knappschaft Vest-Hospital, 45657 Recklinghausen, Germany; 3 Faculty of Medicine, Alexandria University, 21526 Alexandria, Egypt; 4 Faculty of Medicine, Cairo University, 11956 Cairo, Egypt; 5 Tu Lab for Diagnostic Research, Yale School of Medicine, 06510 New Haven, Connecticut, USA; 6 Department of Internal Medicine, College of Medicine, Prince Sattam Bin Abdulaziz University, 16273 Al-Kharj, Kingdom of Saudi Arabia; 7 Faculty of Medicine, Minia University, 61519 Minia, Egypt; 8 Oncological Surgery Unit, Vest-Hospital, 45657 Recklinghausen, Germany; 9 Department of General Surgery, Cairo University, 12613 Giza, Egypt; 10 Department of Oncological Surgery, Helios University Hospital, 42117 Wuppertal, Germany

**Keywords:** CT scan, MRI, Recurrent abdominal hernia, Mesh visualization, Complications, Mesh-related outcomes, Meta-analysis

## Abstract

**Background:**

Recurrent abdominal hernias remain a significant clinical challenge, with relatively high recurrence rates despite prosthetic mesh repair. Accurate imaging modalities are essential to assess mesh positioning and detect complications. Our study aims to compare computed tomography (CT) and magnetic resonance imaging (MRI) for mesh visualization, recurrence detection, and related postoperative outcomes in recurrent hernia patients.

**Methods:**

A systematic review and meta-analysis were conducted, including CT scan or MRI studies, to assess mesh visualization in recurrent hernia cases. A comprehensive search of PubMed, Scopus, Embase, and Web of Science was performed up to July 2024. Data were extracted for mesh visualization, recurrence rates, seroma detection, and reoperation rates. Statistical analysis employed a random-effects model with subgroup analysis for CT and MRI modalities.

**Results:**

A total of 26 studies were included (18 for CT, and 8 for MRI). Recurrence rates were 20% (95% CI: 0–42%) for CT-based studies and 15% (95% CI: 4–26%) for MRI-based studies (p = 0.72). MRI exhibited superior mesh visualization (73%; 95% CI: 42–100%) compared to CT-(48%; 95% CI: 0–100%) (p = 0.44) studies. Seroma detection rates were similar: 12% (95% CI: 4–19%) for CT- and 10% (95% CI: 4–15%) for MRI- (p = 0.65) studies. Reoperation rates were 6% (95% CI: 1–11%) for CT- and 34% (95% CI: 3–66%) for MRI-based studies, showing a non-significant trend (p = 0.08).

**Conclusion:**

CT and MRI offer distinct advantages in detecting mesh-related complications after hernia surgery. CT remains preferred for identifying recurrence and acute complications, while MRI excels in mesh visualization and soft-tissue assessment. Tailored imaging strategies based on clinical scenarios can optimize outcomes and improve postoperative care.

**Supplementary Information:**

The online version contains supplementary material available at 10.1007/s10029-025-03308-9.

## Introduction

Abdominal hernia procedures are among the most common operations performed by general surgeons [1]. Despite prosthetic mesh implants, recurrence rates can reach as high as 40% within five years post-surgery, though these implants have been shown to reduce recurrence rates [2–4]. Recurrent hernias remain a significant clinical challenge, often necessitating precise imaging to evaluate the integrity and placement of the mesh implants [5].

Nowadays, both computed tomography (CT) and magnetic resonance imaging (MRI) are commonly used modalities to detect mesh-related complications [6, 7]. Differences between both modalities are reported in terms of the onset of complications, where CT is thought to be more suitable for acute complications such as bleeding, strictures, or bowel obstruction. In contrast, MRI is preferred for more chronic complications like mesh extrusion, exposure, or abscess formation [8, 9].

CT imaging is a widely used modality due to its availability, accessibility, spectacular high-resolution images, and rapid scanning time, making it a preferred choice in many clinical settings [10–15]. However, a CT scan has some drawbacks, especially its shorthand nature in accurately detecting soft tissue details [13–15]. Additionally, its accuracy diminishes in the presence of significant postoperative changes or scar tissue formation [15–17]. In contrast, MRI has superior capabilities to CT to detect soft tissue details such as mesh visualization, shrinkage, and other soft tissue-related complications, including edema, seroma, granulomas, adhesions, and fibrosis [18].

Given the risk of recurrence, long-term follow-up is often necessary; however, the ionizing radiation from CT scans raises patient safety concerns [19]. MRI offers an advantage by avoiding radiation exposure [19], but its high costs, energy demands, and limited accessibility, reaching only about 10% globally, remain significant drawbacks [20, 21]. MRI also has challenges, including longer scanning times and potential image artifacts that may affect its reliability in specific scenarios [22].

This Meta-analysis addresses the debate between CT’s superior spatial resolution for structural abnormalities and MRI’s enhanced soft-tissue evaluation investigating the clinical outcomes of both modalities, with a focus on mesh visualization and mesh-related complications in recurrent hernias. To the best of our knowledge, no prior meta-analysis has specifically compared CT and MRI for these outcomes in recurrent hernia patients. This study aims to fill this gap in the existing literature.

## Methods

### Data sources and searches

**Fig. 1:**
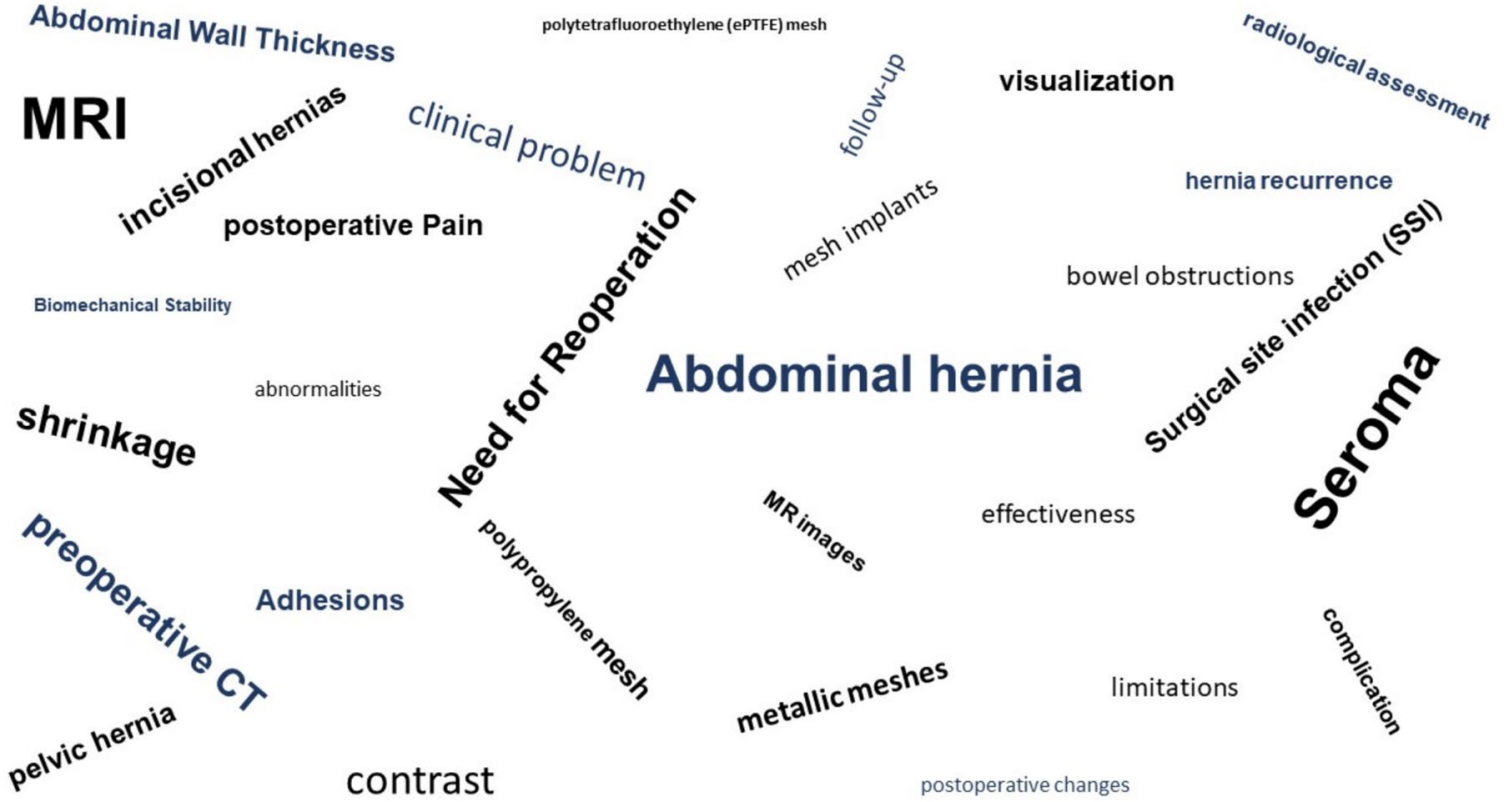
Word cloud

We performed a systematic search of online databases, including PubMed, Scopus, Embase, and Web of Science, from inception up to July 12, 2024. The search terms were designed to capture key concepts related to mesh visualization for detecting recurrent hernias using MRI or CT. Our study commenced on the third of July 2024. The used search strategy was structured as follows:

(Computed Tomography OR CT OR Computed Tomography Angiography OR IVCT OR low-dose CT OR Magnetic Resonance OR Magnetic Resonance Imaging OR MRI OR Magnetic Resonance Angiography OR contrast MRI OR IVMRI OR Diagnosis OR Diagnose) AND (Recurrent hernia OR hernia recurrence OR recurrence of Hernia). Figure 1 presents a word cloud visually representing the search terms and their relevance, emphasizing the primary focus areas of our systematic review.

This study adheres to the Meta-Analysis of Observational Studies in Epidemiology (MOOSE) Checklist [23], and a supplementary table (Supplementary Table 1) is provided to outline compliance with each item. Our meta-analysis adhered to the PICOS (Participants, Intervention, Comparator, Outcomes, and Study Design) framework to ensure methodological rigor. The participant population included patients undergoing imaging for recurrent hernia evaluation, as defined by the inclusion criteria. The interventions assessed were CT and MRI modalities, with direct comparisons between the two imaging techniques. The baseline characteristics of the included studies are presented in Table 1.

**Table 1:** Summary details for Included Studies and Patient Characteristics

Study ID	Country	Period	Sample size	Mean age (± SD)	Gender (Male/Female)	Hernia type	Hernia location	Hernia size (Width, Length, Area)	Mesh position/deformation
MRI Studies									
Fischer, 2007 [24]	Germany	2004–2004	28	58.3 ± 10.4	20/8	Incisional hernia	N/A	N/A	Laparoscopic intraperitoneal onlay mesh / Open repair
Köhler, 2015 [25]	Austria	2013–2014	10	72.9 ± 15.4	6/4	Parastomal hernia	N/A	N/A	Parastomal hernia
Kirchhoff, 2010 [26]	Germany	2005–2008	86	57.5 ± 11.3	62/24	Incisional hernia	N/A	N/A	Laparoscopic / Open repair
Musters, 2016 [27]	Netherlands	2010–2014	10	62.0 ± 11.0	9/6	Perineal hernia	N/A	N/A	Porcine dermal mesh
Paajanen, 2004 [28]	Finland	1997–2002	84	62.0 ± 13.0	30/54	Ventral hernia	N/A	N/A	N/A
Özveri, 2021 [29]	Turkey	2013–2019	7	39.0 ± 10.8	5/7	Inguinal hernia	N/A	N/A	N/A
Hansen, 2015 [5]	N/A	2012–2013	10	61.0 ± 8.5	9/1	Inguinal hernia	N/A	N/A	N/A
van den Berg, 2000 [30]	Netherlands	Not mentioned	10	58.1 ± 9.2	9/1	Inguinal hernia	Anterior abdominal wall	N/A	Unilateral: 7, Bilateral: 3
MRI Studies Summary			245	58.7 ± 12.2	140/55	Multiple hernia types	N/A	N/A	Multiple repair techniques
CT Studies									
Frommer, 2024 [31]	UK	2009–2021	101	54.0 ± 12.8	34/67	Anterior abdominal wall hernia	Anterior abdominal wall	Defect width: 0.991 / Area: 1.000	N/A
Gossios, 2003 [32]	Greece	3.5 years	51	62.0 ± 11.5	13/38	Incisional ventral hernia	Anterior abdominal wall	N/A	N/A
Hietaniemi, 2020 [33]	Finland	2010–2015	165	67.0 ± 10.3	43/122	Paraesophageal hernia	Internal hernia	Size: 30–49% (26); 50–74% (77); 75–99% (22); 100% (32)	N/A
Holihan, 2016 [34]	USA	2010–2011	100	N/A	N/A	Ventral hernia	Anterior abdominal wall	N/A	N/A
Larmark, 2003 [35]	Sweden	1992–1998	404	N/A	N/A	Inguinal hernia	Anterior abdominal wall	N/A	N/A
Liang, 2012 [36]	USA	2000–2010	78	59.0 ± 9.7	N/A	Ventral hernia	Anterior abdominal wall	4.35 cm; 5.8 cm; 27.7 cm^2^	Mesh shift
Kumar, 2022 [37]	UK	2008–2017	188	58.0 ± 10.5	95/93	Ventral hernia	Anterior abdominal wall	N/A	N/A
Pauli, 2014 [38]	USA	2011–2013	29	59.0 ± 8.0	11/18	Ventral hernia	Anterior abdominal wall	17.0 ± 8.0 cm; 410.0 ± 281.0 cm^2^	Midline (22); Lateral (3); Both (4)
Knewitz, 2022 [39]	USA	2011–2020	270	58.0 ± 11.5	147/123	Mixed hernias	Abdominal / inguinal region	N/A	N/A
Schoenmaeckers, 2010 [40]	Netherlands	2001–2010	765	N/A	N/A	Mixed hernias	Anterior abdominal wall	N/A	N/A
Sasse, 2018 [41]	USA	2012–2018	64	59.0 ± 12.0	17/47	Ventral incisional hernia	Anterior abdominal wall	N/A	N/A
Shemyatovsky, 2020 [42]	Russia	2017	40	N/A	N/A	Mixed hernias	Abdominal / inguinal region	N/A	N/A
Van den Dop, 2023 [43]	Europe	2016–2017	84	62.7 ± 12.3	37/24	Midline hernia	Anterior abdominal wall	Recurrence size: 11.8 ± 15.8 cm^2^	Phasix mesh
Beck, 2013 [44]	USA	2010–2012	181	54.0 ± 11.5	123/58	N/A	N/A	Hernia area: 44.6 cm^2^ (range: 0.2–468.3 cm^2^)	N/A
Blair, 2015 [45]	USA	2008–2012	151	55.3 ± 12.5	57/94	Ventral hernia	Midline	8.5 ± 5.0 cm; 7.3 ± 5.6 cm; 178.3 ± 214.0 cm^2^	N/A
Maskal, 2023 [46]	USA	2014–2022	724	N/A	N/A	Ventral incisional hernia	N/A	N/A	N/A
Lin, 1999 [47]	USA	1-year period	33	8 years	9/24	Ventral hernia	Abdominal wall	N/A	N/A
Gutiérrez, 2001 [48]	Spain	2000–2000	50	58.0 ± 10.5	18/32	Incisional hernia	N/A	N/A	N/A
CT Studies Summary			3480	59.3 ± 11.2	697/440	Multiple hernia types	Multiple locations	Various	Multiple repair techniques

Key outcomes included recurrence rates, mesh visualization, seroma detection, and reoperation rates. In this study, the MINORS instrument was applied to evaluate the methodological quality of our studies. This assessment included criteria such as clearly stated aims, consecutive patient inclusion, unbiased endpoint assessment, and adequate follow-up, ensuring a standardized quality evaluation for all studies as per Table 2.

**Table 2:** Assessment of the quality of studies through Methodological Index for Non-Randomized Studies (MINORS) [49]

Study ID	Clearly stated Aim	Consecutive patients	Prospective collection of data	Endpoints	Unbiased assessment of endpoint	Follow-up period	Loss < 5%	Study size	MINORS score
Fischer, 2007 [24]	2	2	2	2	2	2	2	1	15
Köhler, 2015 [25]	2	2	1	2	2	2	2	1	14
Kirchhoff, 2010 [26]	2	2	1	2	2	2	2	1	14
Musters, 2016 [27]	2	2	1	2	2	2	2	1	14
Paajanen, 2004 [28]	2	2	1	2	2	2	2	1	14
Özveri, 2021 [29]	2	2	1	2	2	2	2	1	14
Hansen, 2015 [5]	2	2	1	2	2	2	2	1	14
van den Berg, 2000 [30]	2	2	2	2	2	2	2	1	15
Frommer, 2024 [31]	2	2	1	2	2	2	2	1	14
Gossios, 2003 [32]	2	2	1	2	2	2	2	1	14
Hietaniemi, 2020 [33]	2	2	1	2	2	2	2	1	14
Holihan, 2016 [34]	2	2	1	2	2	2	2	1	14
Larmark, 2003 [35]	2	2	1	2	2	2	2	1	14
Liang, 2012 [36]	2	2	1	2	2	2	2	1	14
Kumar, 2022 [37]	2	2	1	2	2	2	2	1	14
Pauli, 2014 [38]	2	2	1	2	2	2	2	1	14
Knewitz, 2022 [39]	2	2	1	2	2	2	2	1	14
Schoenmaeckers, 2010 [40]	2	2	1	2	2	2	2	1	14
Sasse, 2018 [41]	2	2	1	2	2	2	2	1	14
Shemyatovsky, 2020 [42]	2	2	1	2	2	2	2	1	14
Van den Dop, 2023 [43]	2	2	1	2	2	2	2	1	14
Beck, 2013 [44]	2	2	1	2	2	2	2	1	14
Blair, 2015 [45]	2	2	1	2	2	2	2	1	14
Maskal, 2023 [46]	2	2	1	2	2	2	2	1	14
Lin, 1999 [47]	2	2	1	2	2	2	2	1	14
Gutiérrez, 2001 [48]	2	2	1	2	2	2	2	1	14

Additionally, we ensured alignment with key components of the AMSTAR guidelines, including a comprehensive search, independent review, and quality assessment of included studies. Furthermore, MeSH and Emtree terms were utilized in the database search, and these terms are detailed in Supplementary Table 2.

### Inclusion/Exclusion criteria

We included studies evaluating the recurrence of external hernias in adults, using MRI or CT to visualize biological or synthetic meshes. Eligible studies included full texts, abstracts, and letters published in peer-reviewed journals, limited to the English language. Excluded were studies using ultrasonography or alternative imaging, addressing internal hernias or unrelated conditions, as well as animal studies, case reports, Meta-analyses, and reviews. Studies focusing on internal hernias, including parastomal hernias, were excluded to ensure the scope of the analysis remained on external hernias. The types of included studies are listed in Supplementary Table 3.

### Study selection, and data extraction

Four authors independently conducted the screening process, starting with titles and abstracts, followed by a full-text data extraction. Studies were assessed according to the predefined inclusion and exclusion criteria. Ultimately, 26 studies were selected for inclusion, with 8 studies in the MRI group [5, 24–30] and 18 studies in the CT group [31–48]. Data extraction was then performed to collect baseline characteristics and outcome measures from the included studies, facilitating further analysis.

### Risk of bias

Two reviewers assessed bias in the included studies using the MINORS criteria [49], which rates non-comparative studies on a 0–16 scale and comparative studies on a 0–24 scale. Higher scores reflect lower bias risk. Non-comparative studies were categorized as extremely poor (0–4), low (5–7), fair (8–12), or excellent quality (13–16). Similarly, comparative studies were rated as very poor (0–6), bad (7–10), good (11–15), or excellent quality (16–24).

### Assessment of quality of evidence

The GRADE (Grading of Recommendations Assessment, Development, and Evaluation) system was used. The GRADE system was applied to each outcome included in our meta-analysis [50], considering factors such as study design, result consistency, estimation precision, potential biases, and clinical relevance of the findings.

### Statistical methods

A meta-analysis of proportions was conducted to assess recurrence rates, seroma detection, mesh visualization, and reoperation needs in hernia repair, comparing CT and MRI detection methods. The analysis utilized R Studio (version 2024.09.0, Build 375) with relevant statistical packages [51, 52]. A random-effects model was applied to address heterogeneity among studies [53]. A random-effects model using the DerSimonian-Laird method was applied to account for between-study heterogeneity. Given the substantial heterogeneity observed (I^2^ = 89%, τ^2^ = 0.0085, p < 0.01), the between-study variance component (τ^2^) influenced the weighting calculation, leading to a more uniform distribution of study weights compared to a fixed-effect model. [54–56]. While the study’s weight is substantial in the fixed-effect model (99.2%), the random-effects model appropriately adjusts for the high between-study heterogeneity, resulting in a more balanced weight distribution across studies [54]

Effect sizes were calculated as untransformed proportions, representing event rates in each study. A continuity correction of 0.5 was applied to studies with zero events to ensure valid calculations of proportions and confidence intervals. Heterogeneity was evaluated using the I^2^ statistic, quantifying variability among studies [55].

The use of untransformed proportions with the DerSimonian-Laird estimator effectively addresses between-study heterogeneity while preserving the direct clinical relevance of our findings.

Subgroup analysis compared CT and MRI using the Chi-squared (X2) test [56], and the DerSimonian-Laird method was employed to estimate between-study variance (τ2), with studies weighted by the inverse variance method [57, 58]. Results were displayed in forest plots, showing study proportions, subgroup pooled proportions, and overall proportions with 95% confidence intervals [59]. A p-value < 0.05 was deemed statistically significant for all tests [60]. We conducted Egger’s regression test for funnel plot asymmetry across all outcomes. Egger’s test evaluates the relationship between study size and effect size precision, where a statistically significant p-value (p < 0.05) indicates potential small-study effects or publication bias.

## Results

A total of 26 studies were included, with 8 employing MRI Modalities [5, 24–30] and 18 utilizing CT scans [30–48]. Below, we provide a detailed account of the main outcomes analyzed in our study.

### Search results and study selection

**Fig. 2:**
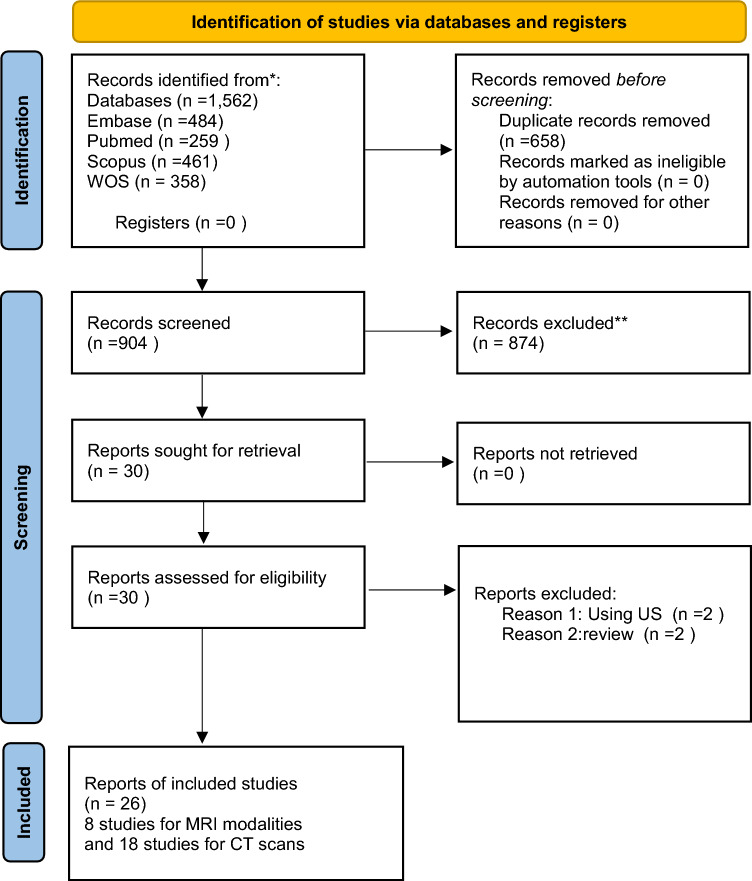
PRISMA flow diagram [61]

A comprehensive database search initially identified 3,124 records. After removing duplicates, 2,466 references remained for title and abstract screening. Following a thorough screening process and full-text review, 26 studies involving a total of 3,725 patients were included in this systematic review and meta-analysis. The PRISMA flow chart of the selection process is shown in Fig. 2 [61].

### Characteristics of the included studies

Table 1 highlights the baseline characteristics of the included studies and the differences between MRI and CT-based studies in recurrent hernia evaluation. Eight MRI studies, comprising 245 patients, were included, compared to 18 CT studies with a total of 3,480 patients. Patient demographics showed differences in gender distribution, with MRI studies reporting 140 males and 55 females (71.8% male) among studies with gender data. CT studies reported 697 males and 440 females (61.3% male) where gender data was available. The mean patient age was comparable between modalities, with MRI patients averaging 58.7 ± 12.2 years and CT patients averaging 59.3 ± 11.2 years.

### Main outcomes

In this study, outcomes were categorized into primary and secondary outcomes to structure the analysis. The primary outcomes included recurrence rates and mesh visualization, as they directly addressed the study’s main objective. The secondary outcomes comprised seroma detection and reoperation rates, providing additional insights into postoperative complications.

### Recurrence Rate

**Fig. 3:**
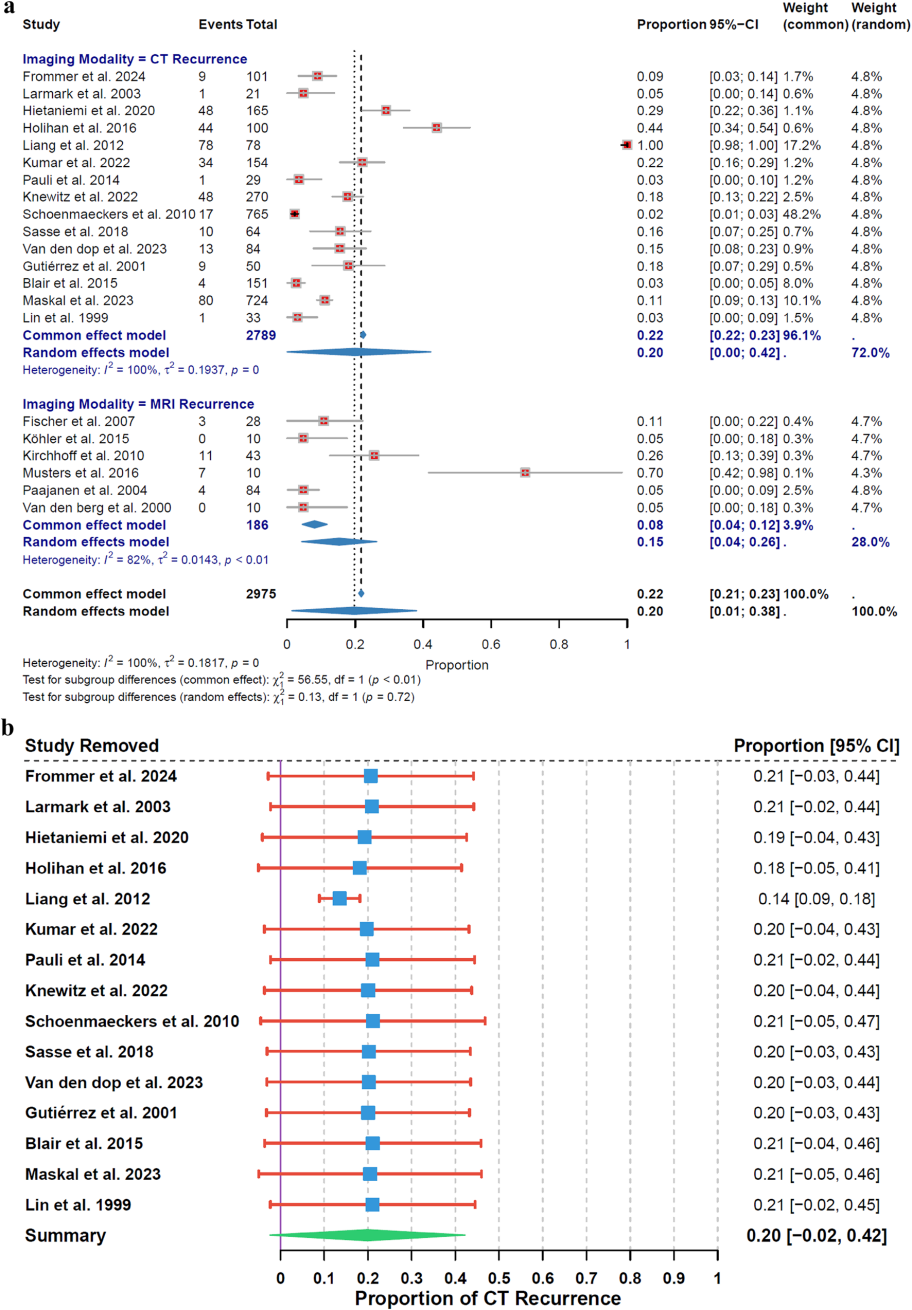

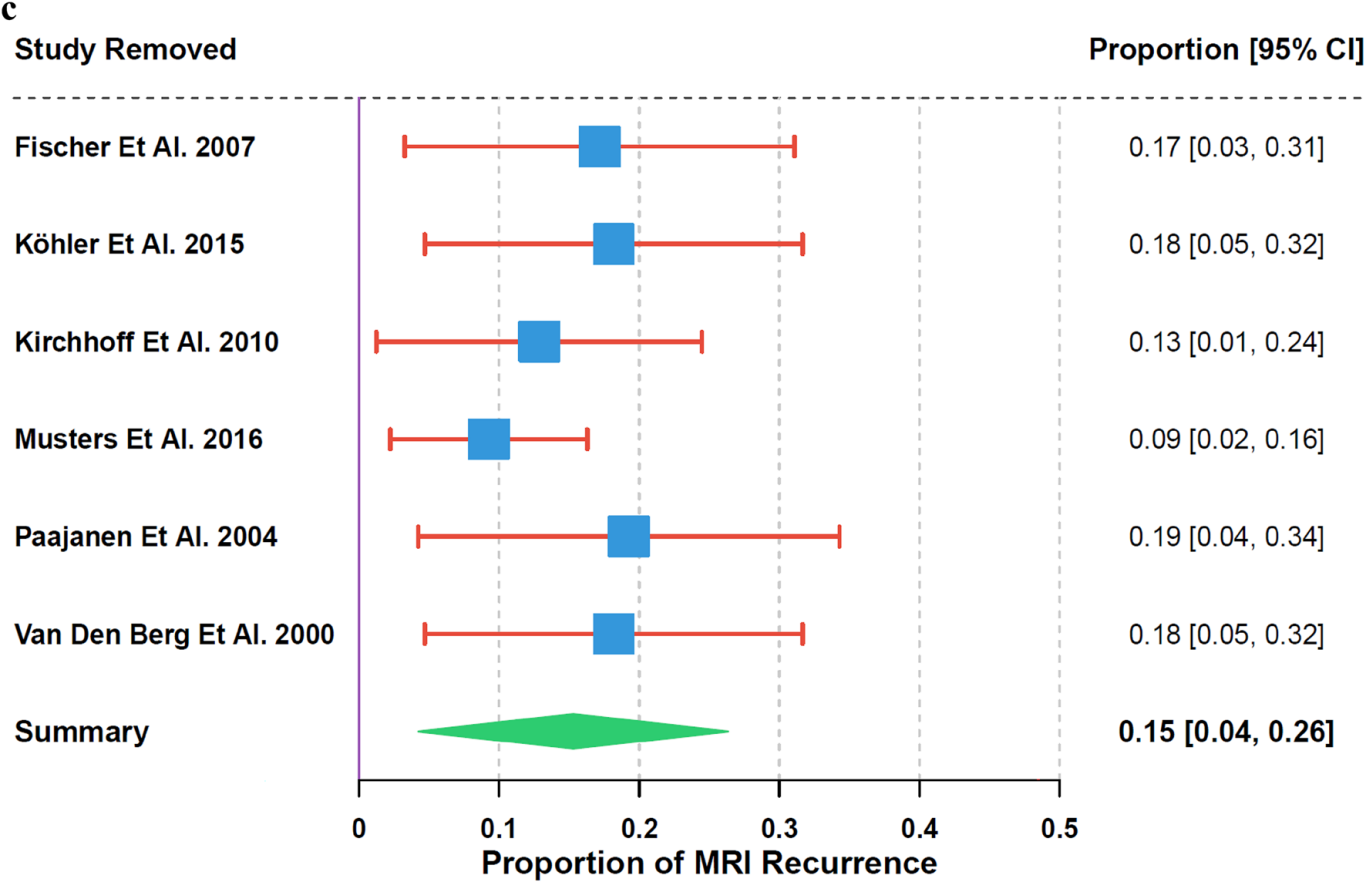
**a** Recurrence Rate by Imaging Modality. **b** Leave − One − Out Sensitivity Analysis for CT Recurrence. **c** Leave − One − Out Sensitivity Analysis for MRI Recurrence

The analysis of recurrence rates using a random-effects model showed an overall estimated proportion of 20% (95% CI: 0% to 42%) for CT and 15% (95% CI: 4% to 26%) for MRI, with high heterogeneity in both (I^2^ = 100%, τ^2^ = 0.1937, p < 0.01 for CT; I^2^ = 82%, τ^2^ = 0.0143, p < 0.01 for MRI). The subgroup analysis, based on 15 studies for CT and 6 studies for MRI, showed no significant variation in recurrence rates between CT and MRI (χ^2^₁ = 0.13, p = 0.72) (Fig. 3a).

Sensitivity analysis highlighted the variability in CT recurrence rates, ranging from 14% (95% CI: 9% to 18%) when Liang et al. 2012 [36] was excluded to 21% (95% CI: −2% to 44%) when Kumar et al. 2022 [37] was removed (Fig. 3b). For MRI, rates ranged from 9% (95% CI: 2% to 16%) when Paajanen et al. 2004 [28] was excluded to 19% (95% CI: 4% to 34%) when Musters et al. 2016 [27] was removed (Fig. 3c).

### Mesh visualization rate

**Fig. 4:**
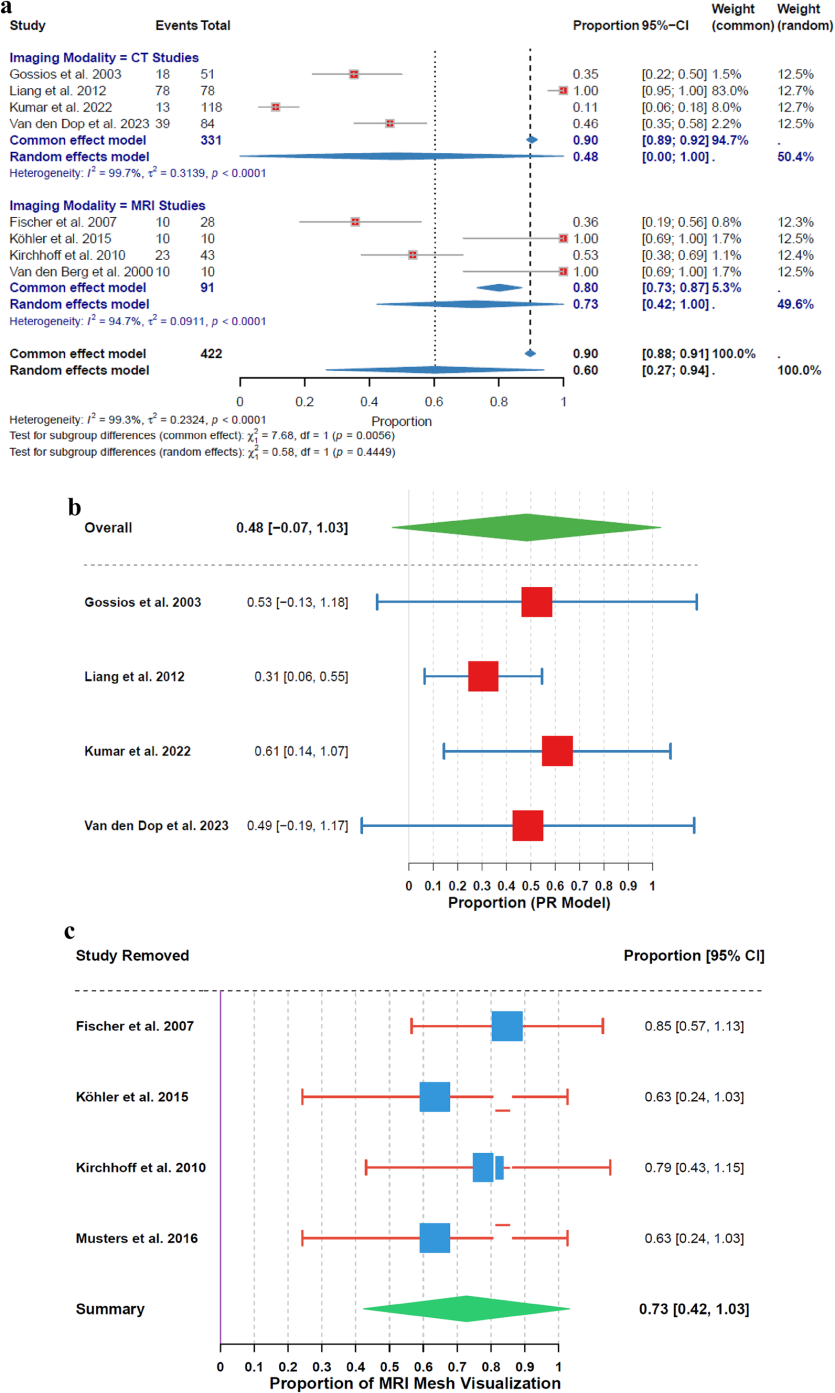
**a** Mesh Visualization Rate by Imaging Modality. **b** Leave − One − Out Sensitivity Analysis for CT Mesh Visualization. **c** Leave − One − Out Sensitivity Analysis for MRI Mesh Visualization

The overall analysis showed a mesh visualization rate of 48% (95% CI: 0% to 100%) for CT and 73% (95% CI: 42% to 100%) for MRI, both with high heterogeneity (I^2^ = 100%, τ^2^ = 0.3139, p < 0.01 for CT; I^2^ = 95%, τ^2^ = 0.0911, p < 0.01 for MRI). Based on 4 studies each for CT and MRI, the subgroup analysis showed no significant variation in mesh visualization rates between the two modalities (χ^2^₁ = 0.58, p = 0.44) (Fig. 4a).

Analysis of CT mesh visualization rates revealed a range from 31% (95% CI: 6% to 55%) when Liang et al. (2012) [36] was excluded, to 61% (95% CI: 14% to 107%) when Kumar et al. (2020) [37] was excluded (Fig. 4b). For MRI, the rates ranged from 63% (95% CI: 24% to 103%) when either Köhler et al. (2015) [25] or Musters et al. (2016) [27] was excluded, to 85% (95% CI: 57% to 113%) when Fischer et al. (2007) [24] was excluded (Fig. 4c).

### Seroma detection rate

**Fig. 5:**
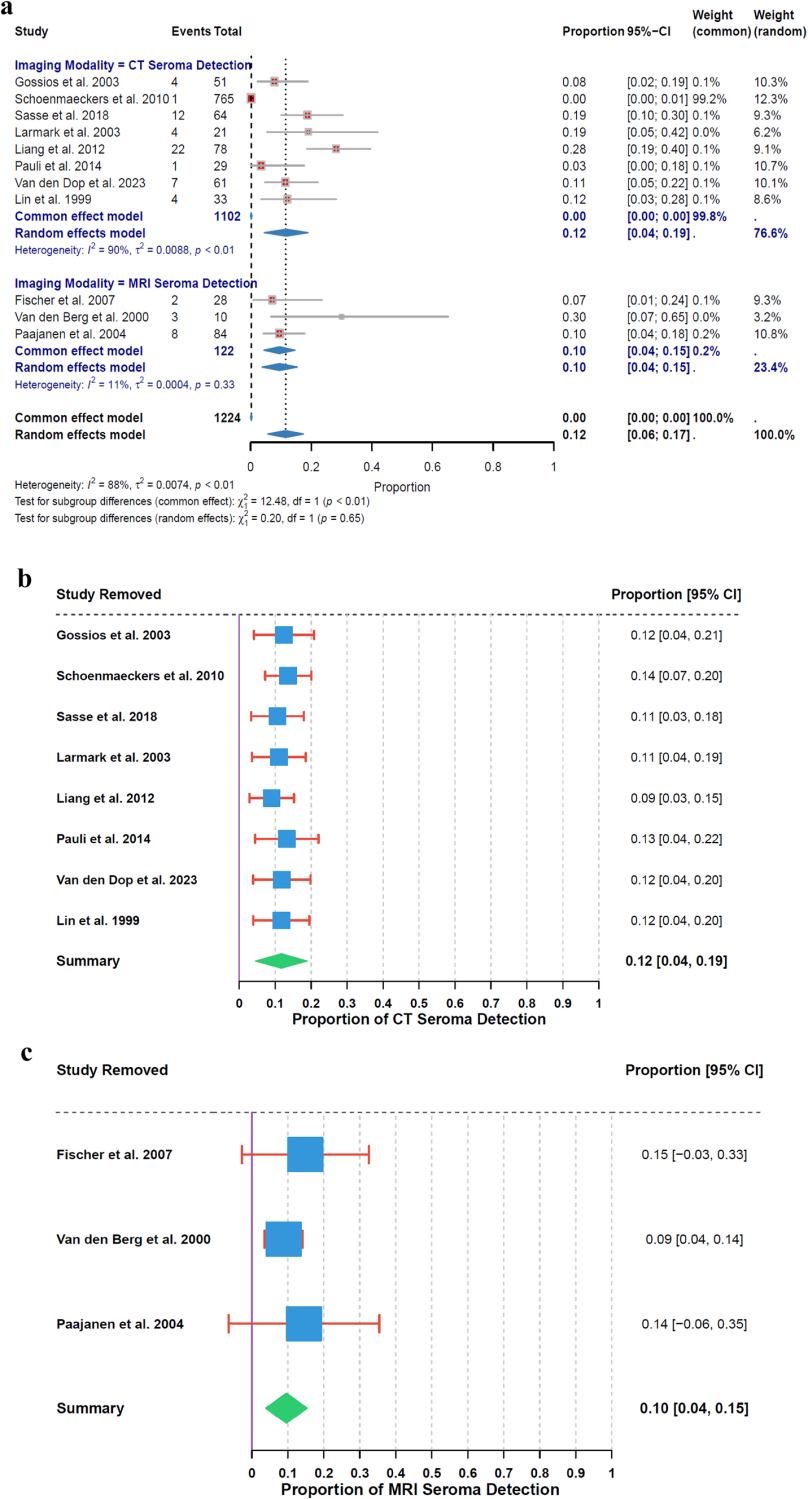
**a** Seroma Detection Rate by Imaging Modality. **b** Leave − One − Out Sensitivity Analysis for CT Seroma Detection. **c** Leave − One − Out Sensitivity Analysis for MRI Seroma Detection

The analysis of seroma detection rates highlighted variations between CT and MRI modalities. The overall estimated seroma detection rate for CT was 12% (95% CI: 4% to 19%), based on 8 studies, with high heterogeneity (I^2^ = 90%, τ^2^ = 0.0088, p < 0.01). In contrast, MRI showed an overall estimated detection rate of 10% (95% CI: 4% to 15%), based on 3 studies, with low heterogeneity (I^2^ = 11%, τ^2^ = 0.0004, p = 0.33). The subgroup analysis revealed no significant differences in seroma detection rates between CT and MRI (χ^2^₁ = 0.20, p = 0.65) (Fig. 5a).

In the sensitivity analysis for CT seroma detection, the highest rate was observed when Liang et al. 2012 [36] was removed (14% [95% CI: 7% to 20%]), while the lowest rate was noted when Sasse et al. 2018 [41] was excluded (11% [95% CI: 3% to 18%]) (Fig. 5b). For MRI seroma detection, the highest rate in the sensitivity analysis was observed when Paajanen et al. 2004 [28] was removed (15% [95% CI: −3% to 33%]), and the lowest when Van den Berg et al. 2000 [30] was excluded (9% [95% CI: 4% to 14%]) (Fig. 5c).

### Reoperation rate

**Fig. 6:**
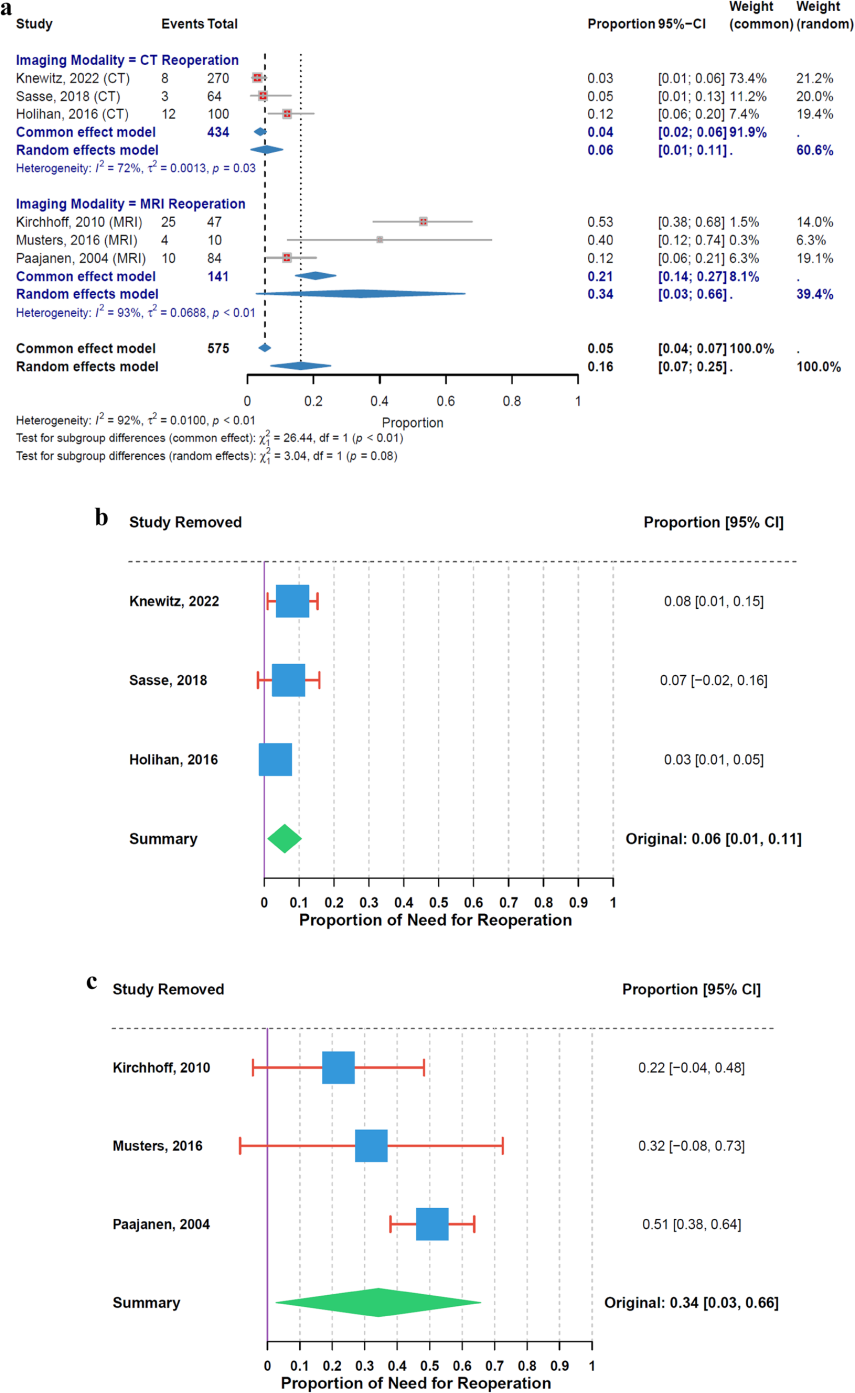
**a** Reoperation Rate by Imaging Modality. **b** Leave − One − Out Sensitivity Analysis for CT Reoperation. 6 Leave − One − Out Sensitivity Analysis for MRI Reoperation

The analysis of reoperation rates revealed variations between CT- and MRI-based studies. The overall estimated reoperation rate for CT was 6% (95% CI: 1% to 11%), based on 3 studies, with moderate heterogeneity (I^2^ = 72%, τ^2^ = 0.0013, p = 0.03). In comparison, MRI had a higher overall estimated reoperation rate of 34% (95% CI: 3% to 66%), also based on 3 studies, with high heterogeneity (I^2^ = 93%, τ^2^ = 0.0688, p < 0.01). Although the test for subgroup differences suggested a trend towards variation in reoperation rates between CT and MRI, it did not reach statistical significance (χ^2^₁ = 3.04, p = 0.08) (Fig. 6a).

During the sensitivity analysis for CT reoperation rates, the highest rate was observed when Holihan et al. (2016) [34] was excluded, resulting in 8% (95% CI: 1% to 15%), while the lowest rate occurred when Sasse et al. (2018) [41] was excluded, yielding 3% (95% CI: 1% to 5%) (Fig. 6b). For MRI reoperation rates, the highest value was recorded when Kirchhoff et al. (2010) [26] was excluded, at 51% (95% CI: 38% to 64%), and the lowest was noted when Paajanen et al. (2004) [28] was excluded, at 22% (95% CI: −4% to 48%) (Fig. 6c).

### Grade summary

The overall certainty of evidence was rated high for recurrence and reoperation rates in both MRI and CT studies, as Egger’s test indicated no significant publication bias (p > 0.05), and the included studies directly addressed the research question. However, for mesh visualization, the certainty of evidence was downgraded to low due to significant publication bias observed in both MRI (p = 0.0082) and CT studies (p = 0.0028), indicating potential small-study effects.

Similarly, the certainty of evidence for CT seroma detection was rated low due to a significant Egger’s test result (p < 0.0001). In contrast, MRI seroma detection rates were supported by high-certainty evidence, as no publication bias was detected (p = 0.7671). Despite the overall precision of the effect estimates, high heterogeneity (I^2^ > 90% in some outcomes) led to further downgrading of evidence for mesh visualization and reoperation rates as shown in supplementary Tables 5, 6.

### Heterogeneity

Significant heterogeneity was noted across CT- and MRI-based studies for hernia evaluation outcomes. For mesh visualization rates, heterogeneity was substantial (I^2^ = 99%, τ^2^ = 0.2324, p < 0.01) (Supplementary eFigure 1a). Subgroup analysis by contrast usage showed persistently high heterogeneity across all subgroups (I^2^ > 88%) (Supplementary eFigure 1a–d), indicating that contrast agent variations alone do not explain the variability. Subgrouping by hernia type revealed differences in heterogeneity levels (Supplementary eFigure 2a–d).

Leave-one-out sensitivity analysis reduced heterogeneity significantly for CT reoperation rates, eliminating it (I^2^ from 72 to 0%) when Holihan et al. (2016) [34] was excluded (Supplementary eFigure 3a). For CT seroma detection, excluding Schoenmaeckers (2010) [40] reduced heterogeneity from 90 to 70% (Supplementary eFigure 3b). In MRI studies, recurrence rate heterogeneity decreased (I^2^ from 82 to 57%) when Musters et al. (2016) [27] was excluded (Supplementary eFigure 4a), and for MRI reoperation rates, excluding Paajanen et al. (2004) [28] reduced heterogeneity to 0% (I^2^ from 93 to 0%) (Supplementary eFigure 4b).

These analyses identified Musters [27], Paajanen [28], and Holihan [34] as key contributors to heterogeneity (Supplementary eFigures 3a, 4a, and b). In contrast, sensitivity analyses for mesh visualization rates and CT recurrence rates showed minimal to no impact on heterogeneity (Supplementary eFigures 3c, d, and 4c).

### Publication bias

Regarding publication bias, funnel plots were generated for mesh visualization, need for reoperation, recurrence rates (Supplementary eFigure 5a–c), and seroma detection (Supplementary eFigure 5d). On visual inspection, asymmetric distribution was noted for mesh visualization and recurrence rates (Supplementary eFigure 5a and c), with several studies falling outside the expected funnel boundaries, indicating possible publication bias. For the need for reoperation and seroma detection rates (Supplementary eFigure 5b and d), although some asymmetry was observed, most studies fell within the expected funnel boundaries, suggesting less likelihood of publication bias for these outcomes. In order to assess the publication bias using Egger’s test, the p-values for each outcome were as follows: recurrence in MRI studies (p = 0.4203) and CT studies (p = 0.4777) indicated no significant publication bias. For mesh visualization, significant evidence of publication bias was found in both MRI studies (p = 0.0082) and CT studies (p = 0.0028). The seroma outcomes showed no significant publication bias for MRI studies (p = 0.7671), whereas CT studies showed strong evidence of publication bias (p < 0.0001). Lastly, for the need for reoperation, both MRI (p = 0.8837) and CT (p = 0.6865) studies demonstrated no significant publication bias as per Supplementary Table 5, 6.

## Discussion

The selection of an imaging modality after hernia surgery depends on the clinical context and type of complication, highlighting the importance of a tailored approach to postoperative care [62]. Early detection of complications through appropriate imaging can guide decisions between conservative and surgical management, reducing morbidity [63].

CT scans remain the “gold standard” for diagnosing abdominal hernias, particularly in recurrence cases, due to their detailed imaging of muscular and fascial layers, defect detection, and ability to differentiate herniated contents from muscle atrophy [14]. Supported by 18 studies and 3,480 patients in this meta-analysis, CT’s reliability in recurrent hernia diagnosis is well-established.

While ultrasonography is useful as a preliminary tool, it cannot often fully assess recurrent hernias or detect mesh displacement, emphasizing CT’s superiority for detailed and effective evaluation [64].

This systematic review and meta-analysis, the first to evaluate MRI’s performance in mesh visualization, demonstrated its superiority over other modalities. These findings align with Weyhe et al. [65], who used a 3D reconstruction approach for MRI-visible meshes, concluding that standard MRI images are sufficient for analysis without significant changes in visualization. Conversely, CT outperformed other modalities in detecting recurrence rates, consistent with Ghafoor et al. (2023) [66], which highlighted CT’s effectiveness in identifying and characterizing inguinal hernias and recurrence. These results highlight the complementary roles of MRI and CT, with MRI excelling in mesh visualization and CT in recurrence detection.

The 100% visualization rate for CT reported by Liang et al., 2012 [36], is a noteworthy outlier in our analysis. While this result highlights the potential for exceptional performance under specific conditions, it may also disproportionately influence the pooled visualization rate for CT. Factors such as patient selection, imaging protocols, and study design could contribute to this high rate and may not represent broader clinical practice. The MRI data from Köhler et al. [25]and Musters et al. [27] present unique findings that may influence the overall analysis. These studies reported notably high visualization rates, likely due to specific imaging protocols or the use of MRI-visible meshes. While these results underscore MRI’s potential for superior soft-tissue evaluation, they may also disproportionately impact the pooled estimates in our meta-analysis. This highlights the variability in study methodologies and the potential for certain studies to skew aggregated outcomes.

These findings underscore the importance of interpreting pooled results in the context of individual study variability and the potential for outliers to skew overall estimates.

Postoperative radiological findings are influenced by factors such as mesh material, density, thickness, and inflammatory response. Commonly used meshes, including polypropylene (PP) and expanded polytetrafluoroethylene (ePTFE), were analyzed in two studies, with Fischer et al. [24], and Kirchhoff et al. [26] noting ePTFE meshes were clearly visible on MRI.

The effectiveness of imaging in mesh visualization can be influenced by the type and brand of mesh used. Upon reviewing the included studies, we found that of the 18 CT modality studies, only 5 used a single type of mesh, 9 did not comment on the type of mesh used, and 4 used a mix of meshes. Similarly, among the 8 studies using MRI modality, 3 employed modified PVDF iron-loaded meshes, 2 used polypropylene meshes, 2 used dual meshes, and 1 study used the Strattice biological mesh. This variability precluded a detailed subgroup analysis.

As highlighted in the literature, certain types of meshes, such as those loaded with tiny iron particles embedded in the base material (e.g., modified PVDF meshes and PTFE composite meshes), Proline meshes, and dual gore meshes, can be detected on both modalities. Conversely, Ventralex and Parietex meshes, as well as Proline meshes, are primarily visible on CT, while intra meshes with silicone layers are better visualized on MRI, as noted in the study by Rakic et al., 2013 [67]. This underscores the need for future research to systematically evaluate imaging performance based on mesh material and brand.

Additionally, mesh placement (preperitoneal or intraperitoneal) and the presence of staples affect imaging outcomes [68, 69], though insufficient data in the included studies prevented a detailed analysis of these factors.

Mesh deformation, such as shrinkage, may contribute to mesh-related pain. Modern MRI-visible meshes enable monitoring of mesh positioning and time-dependent changes in mesh characteristics and complications. Özveri et al. (2021) [29] demonstrated that iron-loaded MRI-visible meshes effectively visualize mesh deformation and surrounding tissue reactions, aiding in the evaluation of postoperative complications.

Kirchhoff et al. (2010) [26] highlighted the advantages of functional cine MRI for detecting intra-abdominal adhesions and recurrent hernias, offering dynamic evaluations beyond CT’s capabilities. These advancements, combined with MRI-visible meshes, provide detailed insights into mesh behavior and tissue responses, improving postoperative care and complication management [70].

The seroma detection rate for CT was comparable to that of MRI, although the number of CT-based studies analyzing seroma detection was more than double the number of MRI-based studies. Functional MRI, on the other hand, proved to be highly effective for evaluating and assessing implanted meshes following hernia repair [37, 66].

Regarding the Schoenmaeckers et al. (2010) study, we acknowledge its substantial impact on our analysis due to its large sample size (n = 765) and notably low seroma detection rate. This can be attributed to their standardized detection protocols, strict definition of clinically significant seroma, consistent timing of assessment, and uniform surgical technique. These factors likely contributed to the lower reported incidence, which may have influenced the overall pooled estimates [40].

The analysis of reoperation rates revealed notable differences, with CT showing a lower overall rate of 6% compared to 34% for MRI. Holihan et al. (2016) [34] supported the lower CT rates, highlighting its effectiveness in identifying complications that reduce the need for surgery. Conversely, Kirchhoff et al. (2010) [26] reported higher reoperation rates for MRI, attributing this to its superior ability to detect complications requiring surgical management. These findings emphasize CT’s role in early complication detection and MRI’s strength in detailed soft-tissue evaluation. The choice of modality should be tailored to clinical needs, as outlined in Table 3.

**Table 3:** CT and MRI Charachterstics

Feature	CT	MRI
Primary Strength	Detecting recurrence and acute complications	Superior mesh visualization and soft-tissue assessment
Use Case	Preferred in emergencies	Suitable for elective evaluations
Speed	Rapid imaging	Longer scanning time
Radiation	Involves ionizing radiation	No radiation exposure
Soft Tissue Assessment	Less effective	Excellent soft tissue visualization
Cost and Accessibility	More affordable and widely available	Higher cost, limited availability
Complications Detected	Recurrence, bleeding, bowel obstruction, incarceration	Mesh shrinkage, seroma, migration, fibrosis, adhesions
Recommended Usage	Acute complications, structural abnormalities	Postoperative mesh evaluations, detailed soft tissue analysis

**Fig. 7:**
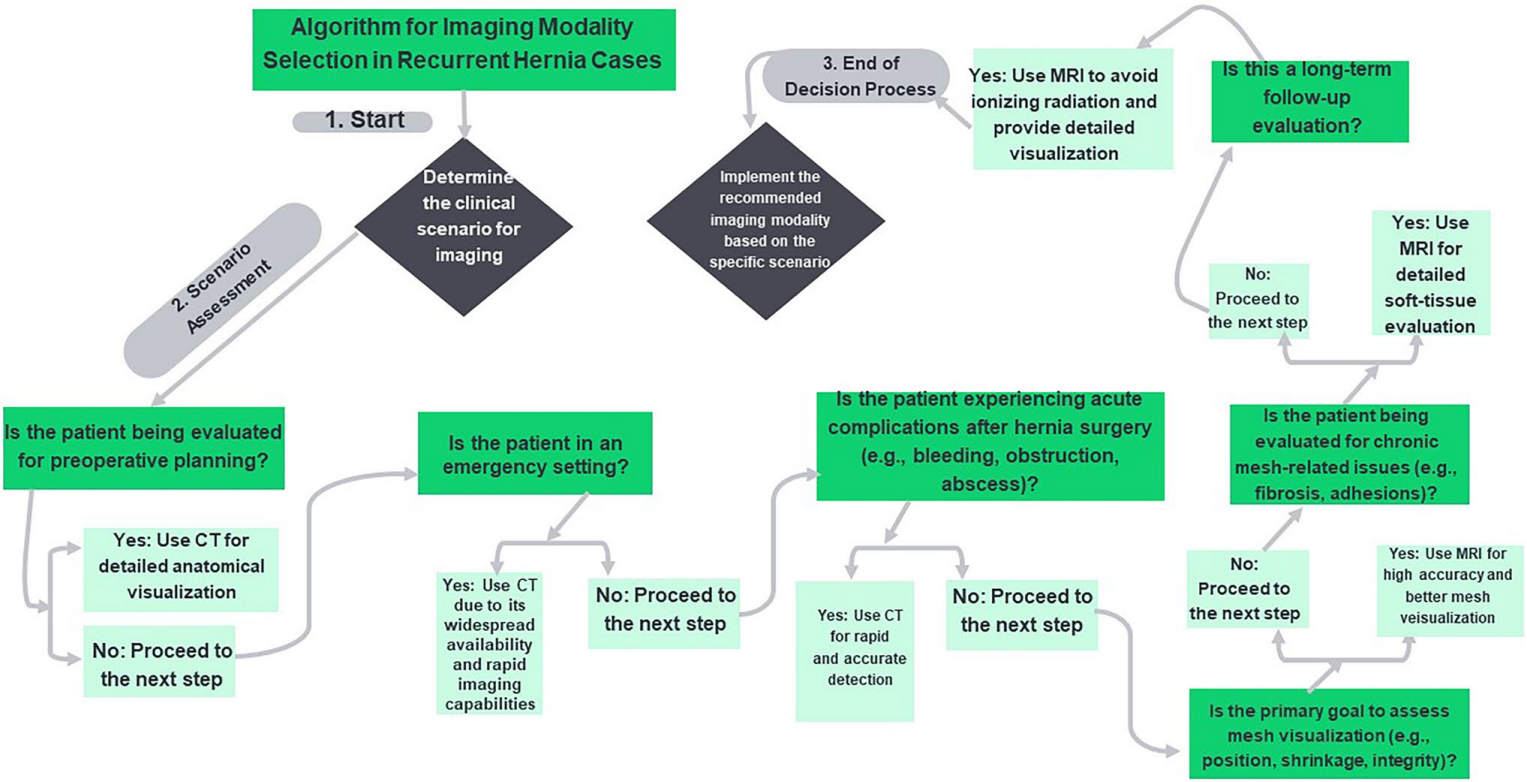
Flowchart for clinical scenario

Figure 7 provides a concise summary of recommendations for selecting CT or MRI based on specific clinical scenarios, highlighting the strengths of CT in acute and emergency settings and the mesh visualization and superior soft-tissue evaluation capabilities of MRI for long-term follow-up and chronic complications as well. These findings are further detailed in Supplementary Table 4.

Our analysis highlights an imbalance between CT and MRI data, with CT-based studies being more prevalent. This disproportion may overrepresent CT’s strengths while limiting the generalizability of MRI findings. Additional MRI-focused studies are needed to provide a balanced comparison, validate its advantages in mesh visualization, and strengthen pooled analyses for comprehensive evaluations.

### Addressing heterogeneity

Heterogeneity posed a significant challenge in analyzing CT-based outcomes due to variability in study designs, populations, and methodologies. CT recurrence rates showed high overall heterogeneity (100%), with a modest reduction to 98% in non-contrast studies. Subgrouping by hernia type further reduced heterogeneity, notably to 77% for incisional hernias and 93% for ventral hernias, though it remained high for inguinal (100%) and other hernia types (96%).

Similarly, for CT mesh visualization, heterogeneity was 100% overall but decreased to 94% with intravenous contrast. Subgrouping by hernia type led to significant reductions, particularly for inguinal hernias (0%), ventral hernias (91%), and incisional hernias (96%). For CT seroma detection, overall heterogeneity was 90% but dropped to 70–78% depending on hernia type. Unexpectedly, subgrouping non-contrast studies for CT reoperation rates increased heterogeneity to 90%, while grouping by hernia type reduced it to 67–98%. These findings highlight the importance of standardized imaging protocols and consistent reporting to reduce variability in future research.

Given the significant heterogeneity in our analysis, we believe the random-effects model provides a more appropriate representation of the true uncertainty in our estimates [71].

## Limitation

Our study is limited by the inability to include mesh shrinkage and biomechanical stability parameters due to insufficient data. Additionally, the small number of patients in MRI-based studies and significant heterogeneity across the included studies restrict the generalizability of our findings. Limited data on mesh material and brand also prevented a meaningful subgroup analysis, and the lack of information on the location of the mesh, whether placed preperitoneally or intraperitoneally, further constrains the scope of our analysis. Furthermore, no randomized controlled trials (RCTs) were included as none met the inclusion criteria for this specific research question. While the observational studies included were rigorously assessed for quality using validated tools such as the MINORS and GRADE criteria, the absence of RCTs may limit the overall level of evidence. Future research should prioritize standardized methodologies, larger high-quality studies, and well-designed RCTs to enhance reliability and applicability in this field.

## Conclusion

Radiological assessment remains critical for monitoring hernia repair outcomes, with CT and MRI offering distinct advantages. CT remains a preferred modality for detecting acute complications and recurrence due to its accessibility and rapid imaging capabilities, making it ideal for emergency settings. Conversely, MRI excels in mesh visualization and soft-tissue assessment, rendering it more suitable for elective evaluations and long-term follow-up, especially for young patients. However, MRI’s limitations include higher costs, longer scan times, and restricted availability.

The choice between CT and MRI should be guided by clinical needs, mesh characteristics, and available resources. Addressing the current limitations, such as insufficient data on mesh shrinkage and biomechanical stability, will help refine imaging strategies and improve patient care in future studies.

## Supplementary Information

Below is the link to the electronic supplementary material.

Supplementary file1 (DOCX 2461 KB)

Supplementary file2 (DOCX 18 KB)

Supplementary file3 (DOCX 15 KB)

Supplementary file4 (DOCX 14 KB)

Supplementary file5 (DOCX 16 KB)

Supplementary file6 (DOCX 13 KB)

Supplementary file7 (DOCX 13 KB)

## Data Availability

All data analysed during this study are included in this published article and its supplementary information files.

## Abbreviations

CT: Computed tomography
ePTFE: Expanded polytetrafluoroethylene
IVCT: Intravenous contrast Computed tomography
MRI: Magnetic resonance imaging
n: Number
PP: Polypropylene
PVDF: Polyvinylidene flouride
Vs: Versus
