# Supplementary material for: Conflict resolution of the beams: CT vs. MRI in recurrent hernia detection: a systematic review and meta-analysis of mesh visualization and other outcomes
Source: Hernia. 2025 Mar 28;29(1):127. doi: 10.1007/s10029-025-03308-9 (PMC11953100; doi:10.1007/s10029-025-03308-9)

**Supplementary Figures (Online only material)**

Supplementary Figure 1a) Mesh Visualization Rate by Contrast Agent Usage – [Page16](#Page24)

Supplementary Figure 1b) Recurrence Rate by Contrast Agent Usage –[Page17](#Page25)

Supplementary Figure 1c) Reoperation Rate by Contrast Agent Usage –[Page18](#Page26)

Supplementary Figure 1d) Seroma Rate by Contrast Agent Usage – [Page19](#Page27)

Supplementary Figure 2a) Mesh Visualization Rate by Hernia Type -[Page20](#Page28)

Supplementary Figure 2b) Reoperation Rate by Hernia Type - [Page21](#Page29)

Supplementary Figure 2c) Recurrence Rate by Hernia Type – [Page22](#Page30)

Supplementary Figure 2d) Seroma Rate by Hernia Type – [Page23](#Page31)

Supplementary Figure 3a) Leave−One−Out Sensitivity Analysis (Random−Effects) for CT Reoperation Rates – [Page24](#Page32)

Supplementary Figure 3b) Leave−One−Out Sensitivity Analysis (Random−Effects) for CT Seroma Rates – [Page25](#Page33)

Supplementary Figure 3c) Leave−One−Out Sensitivity Analysis (Random−Effects) for CT Recurrence Rates – [Page26](#Page34)

Supplementary Figure 3d) Leave−One−Out Sensitivity Analysis (Random−Effects) for CT Mesh Visualization Rates – [Page27](#Page35)

Supplementary Figure 4a) Leave−One−Out Sensitivity Analysis (Random−Effects) for MRI Recurrence Rates – [Page28](#Page36)

Supplementary Figure 4b) Leave−One−Out Sensitivity Analysis (Random−Effects) for MRI Reoperation Rates – [Page29](#Page37)

Supplementary Figure 4c) Leave−One−Out Sensitivity Analysis (Random−Effects) for MRI Mesh Visualization Rates - [Page30](#Page38)

Supplementary Figure 5a) Funnel Plot: Mesh Visualization by Imaging Modality - [Page31](#Page39)

Supplementary Figure 5b) Funnel Plot: Need for Reoperation by Imaging Modality – [Page32](#Page40)

Supplementary Figure 5c) Funnel Plot: Recurrence Across Imaging Modalities – [Page33](#Page41)

Supplementary Figure 5d) Funnel Plot: Seroma Incidence by Imaging Modality – [Page34](#Page42)

Supplementary Figure 1a) Mesh Visualization Rate by Contrast Agent Usage


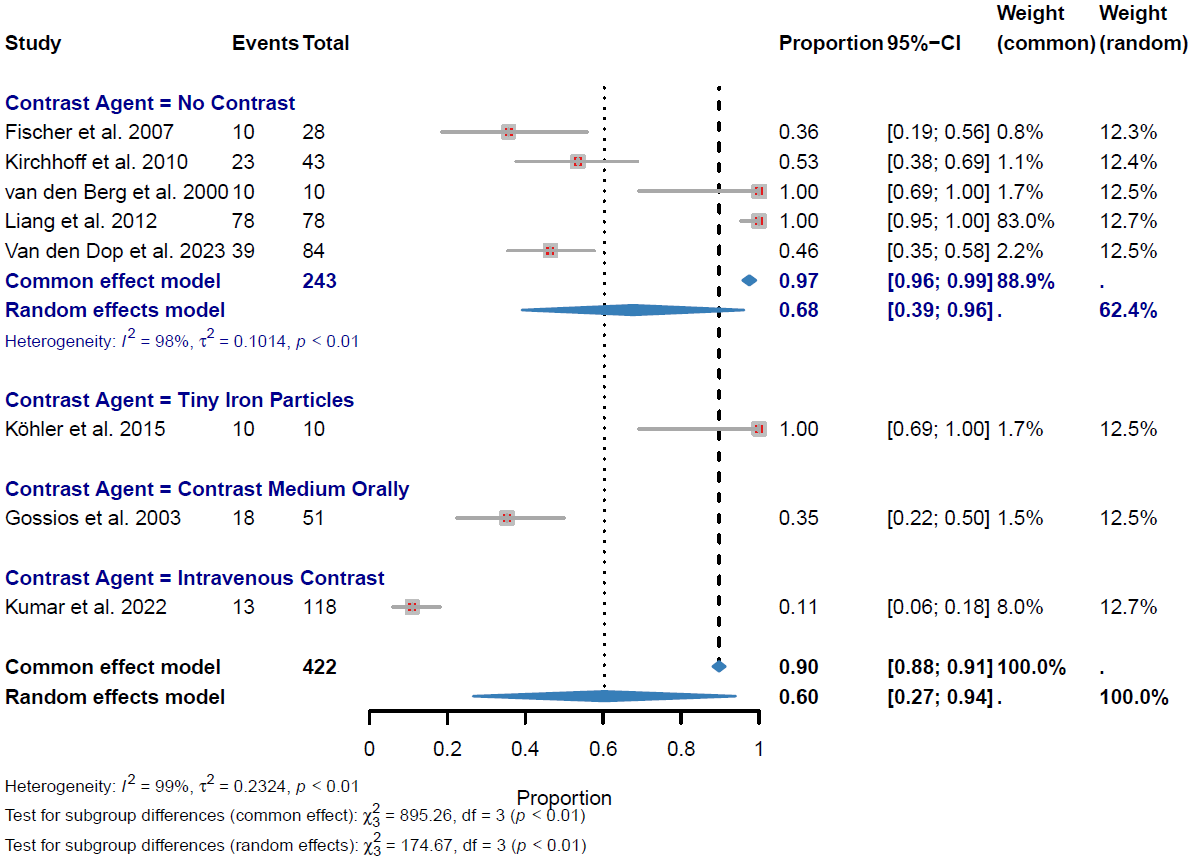


Supplementary Figure 1b) Recurrence Rate by Contrast Agent Usage


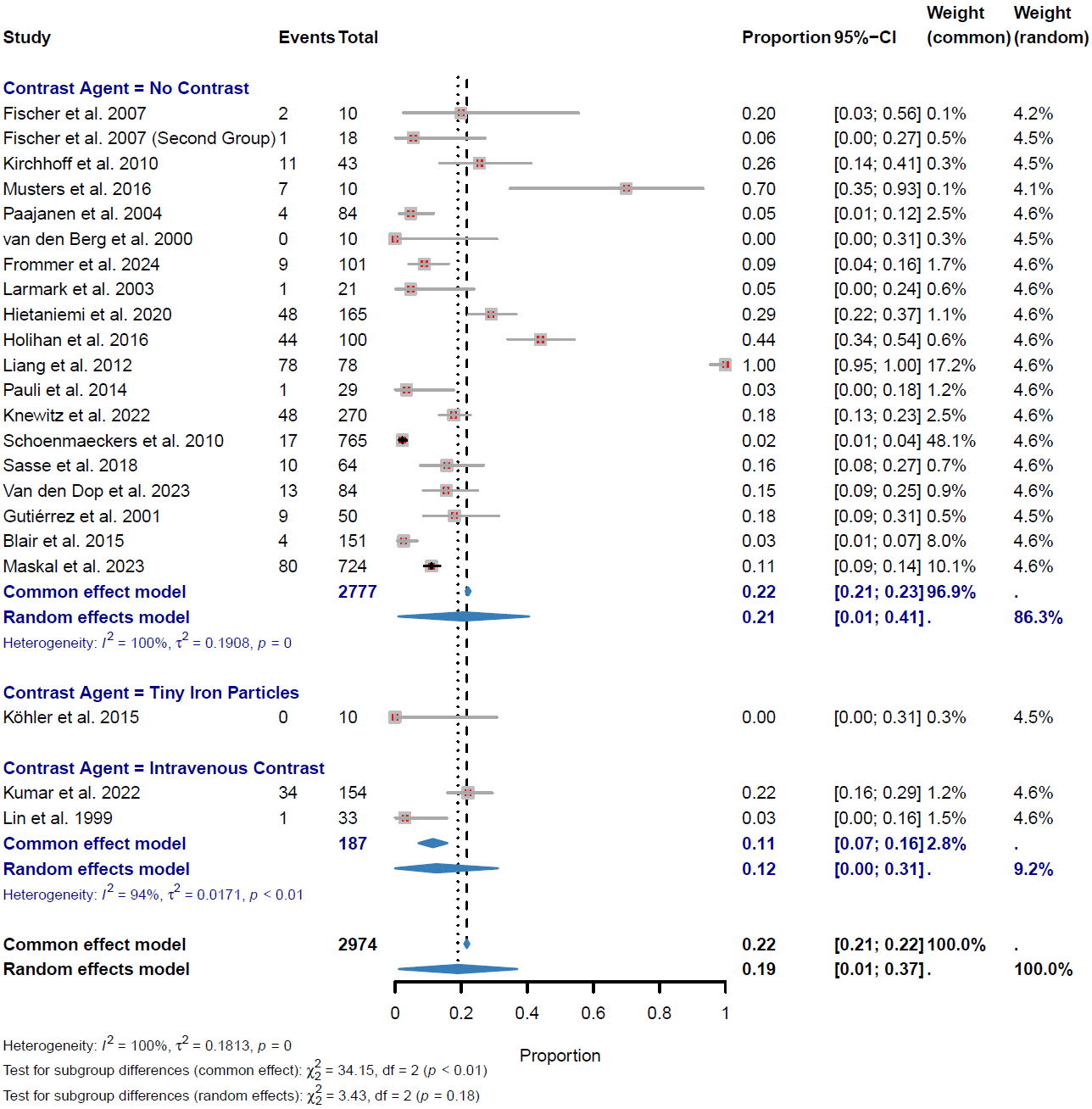


Supplementary Figure 1c) Reoperation Rate by Contrast Agent Usage


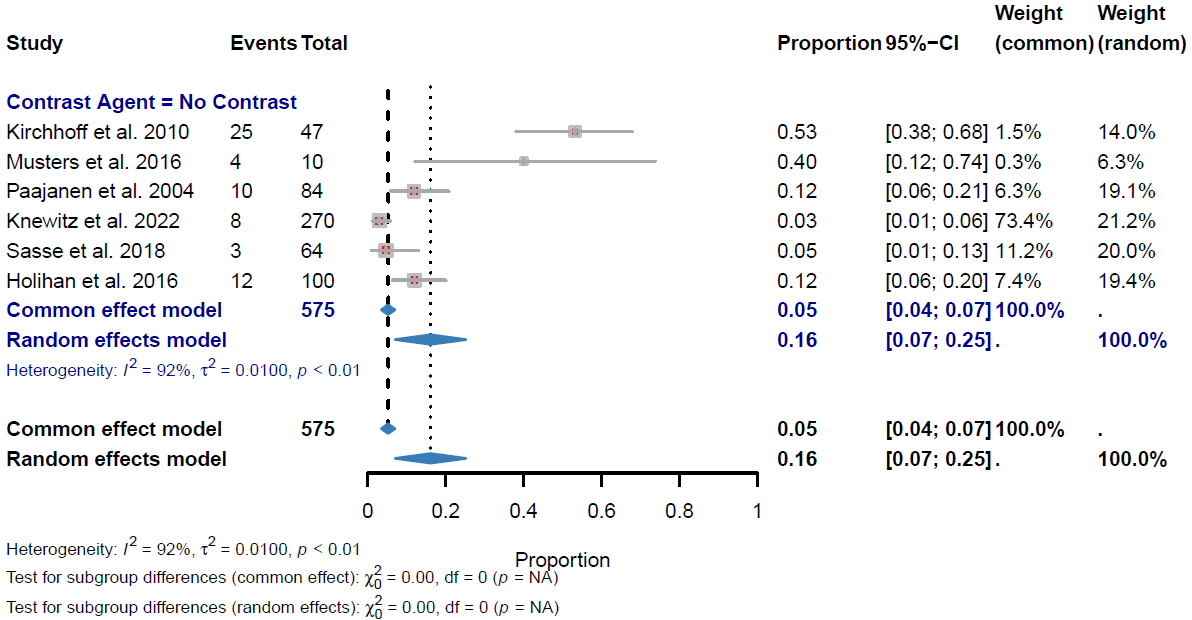


Supplementary Figure 1d) Seroma Rate by Contrast Agent Usage


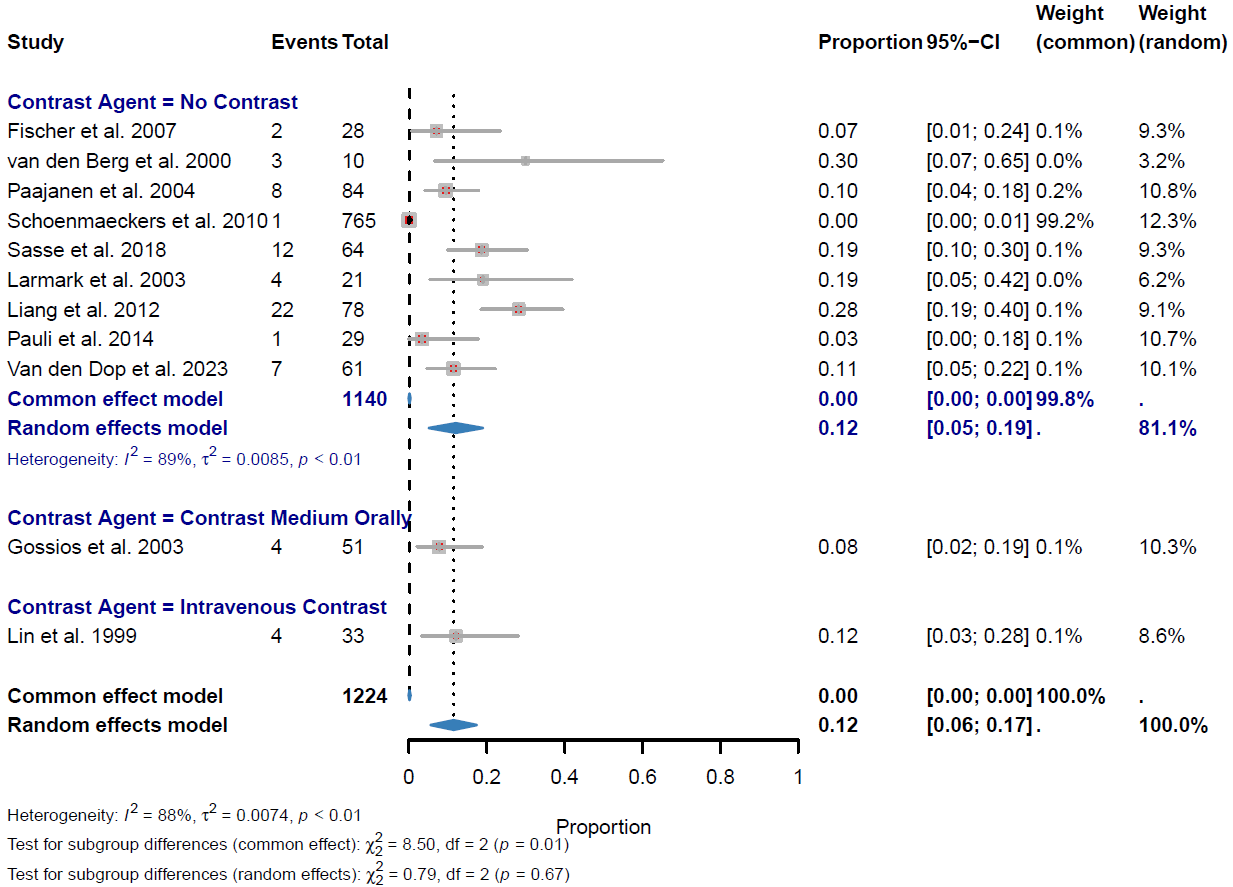


Supplementary Figure 2a) Mesh Visualization Rate by Hernia Type


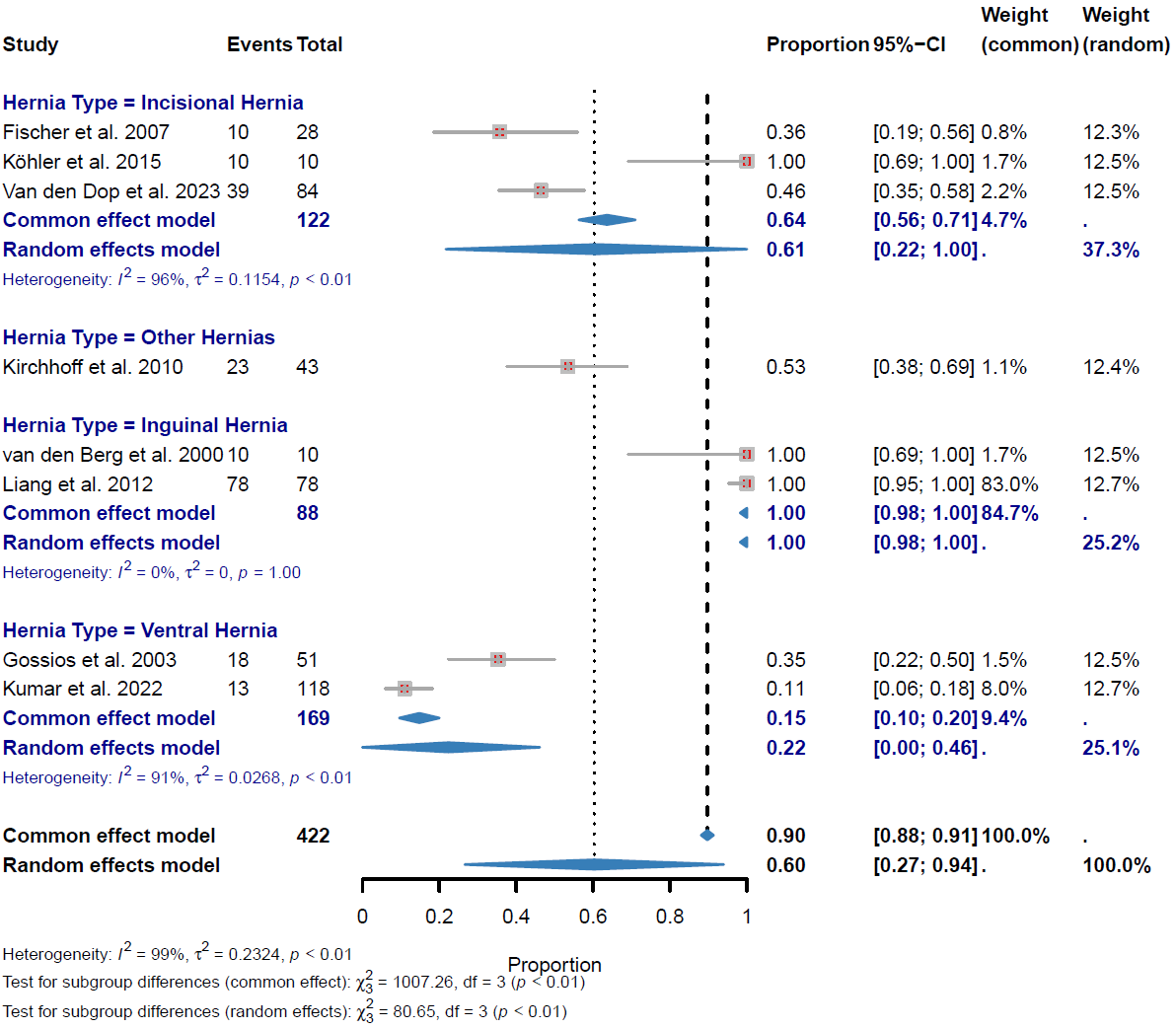


Supplementary Figure 2b) Reoperation Rate by Hernia Type


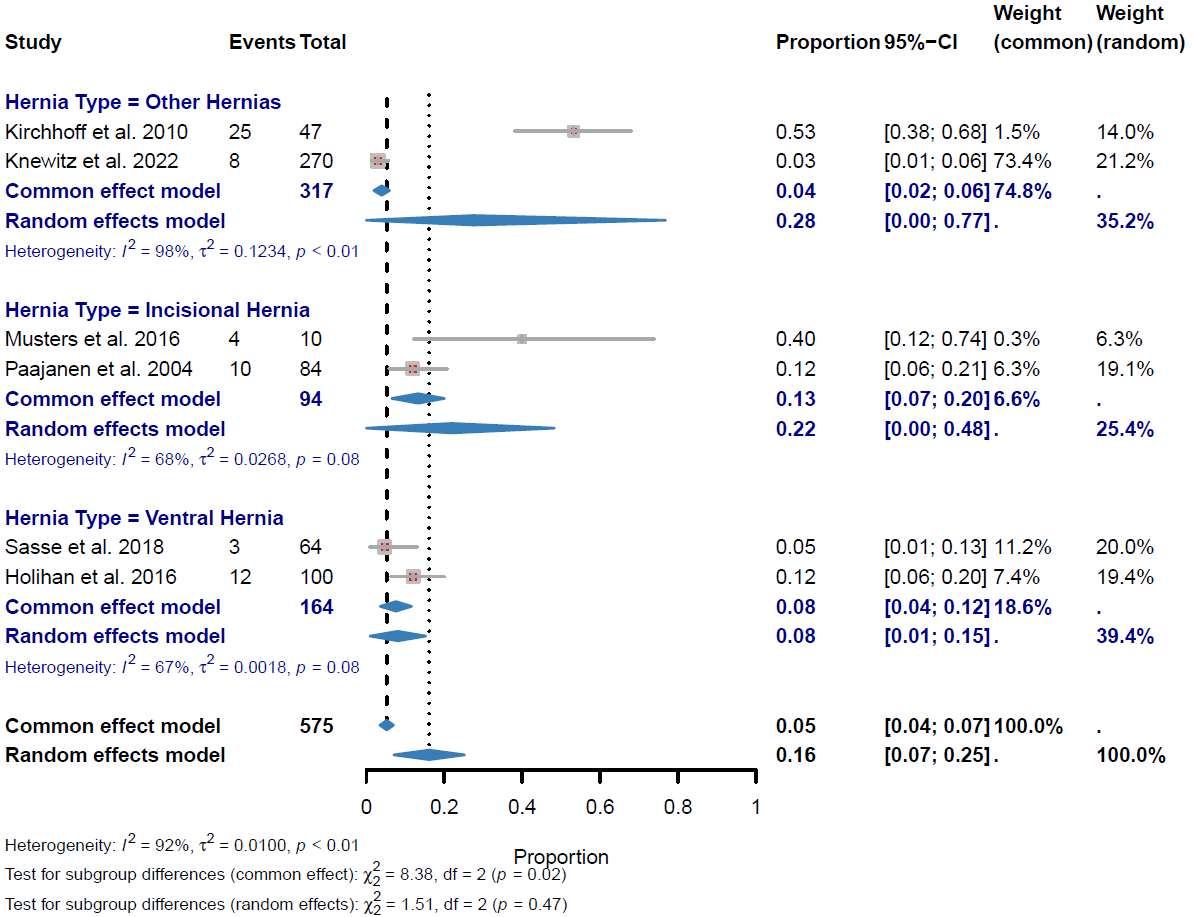


Supplementary Figure 2c) Recurrence Rate by Hernia Type


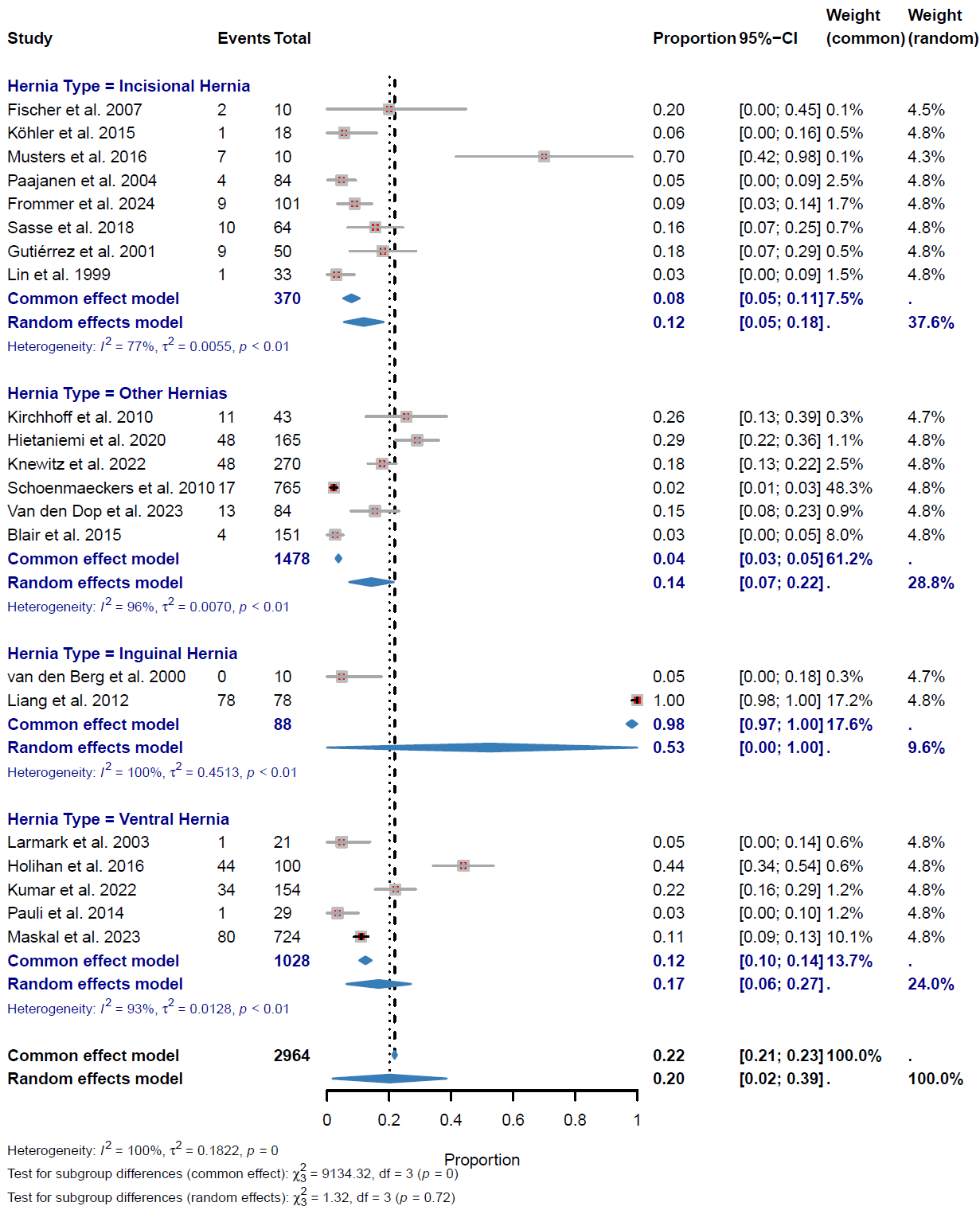


Supplementary Figure 2d) Seroma Rate by Hernia Type


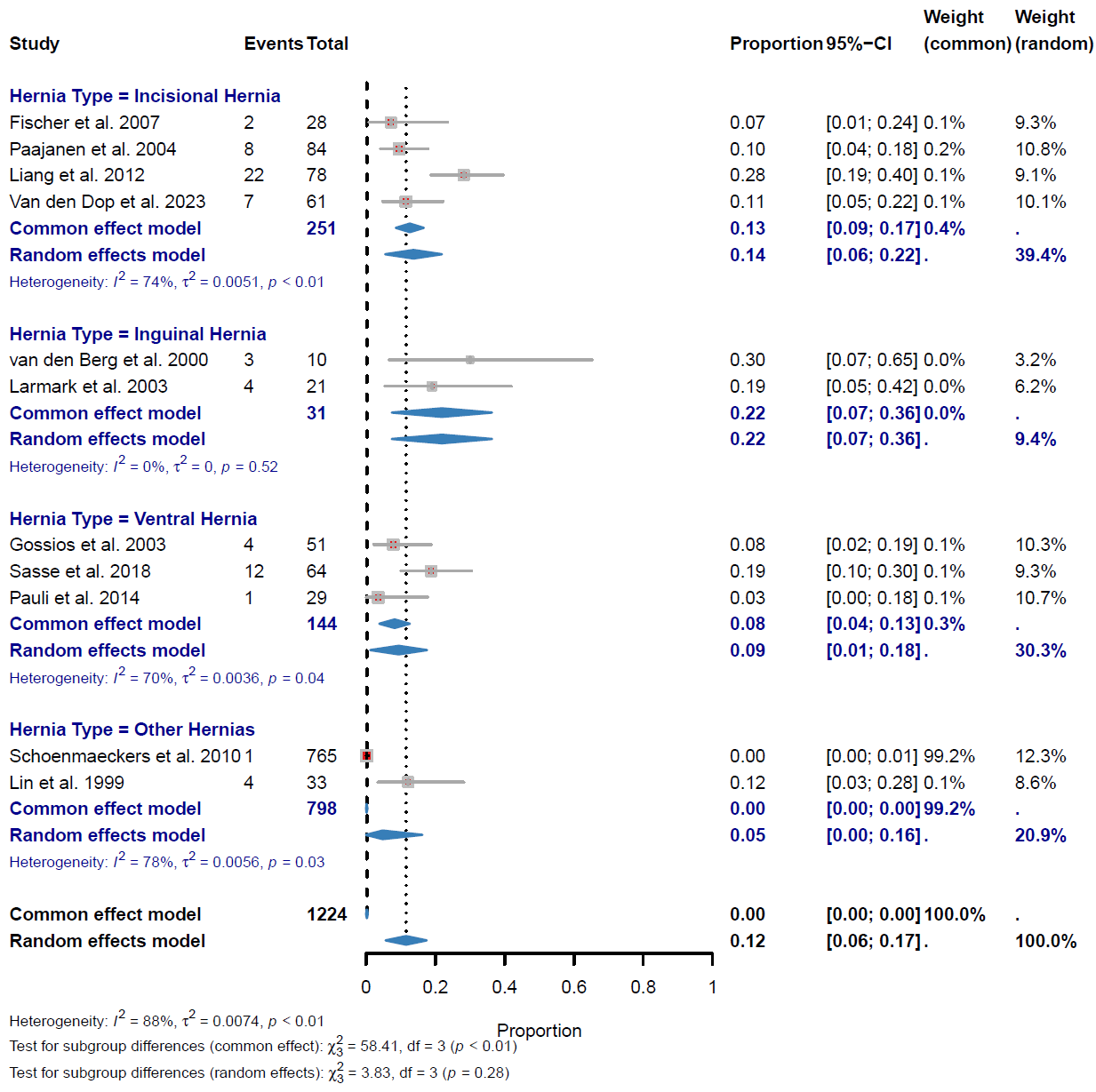


Supplementary Figure 3a) Leave−One−Out Sensitivity Analysis (Random−Effects) for CT Reoperation Rates


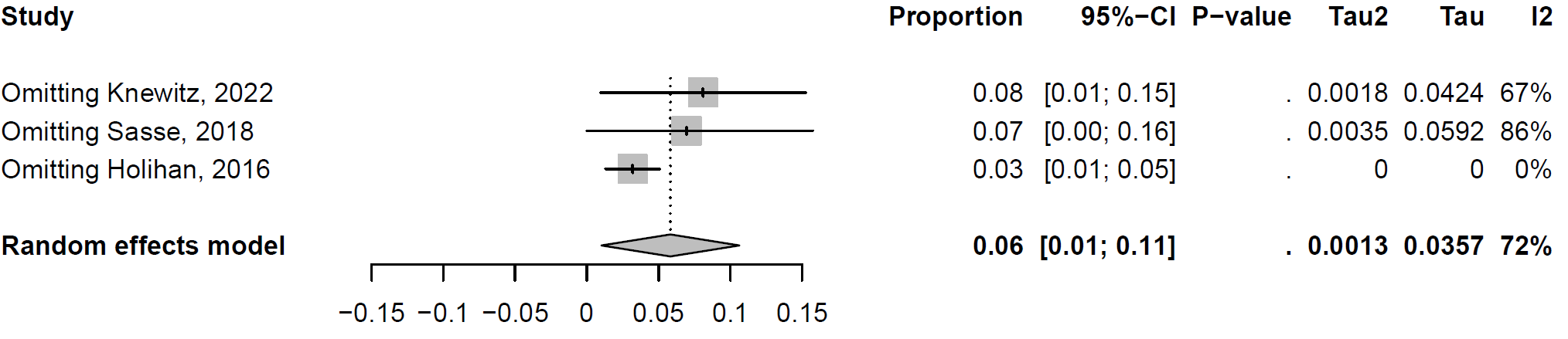


Supplementary Figure 3b) Leave−One−Out Sensitivity Analysis (Random−Effects) for CT Seroma Rates


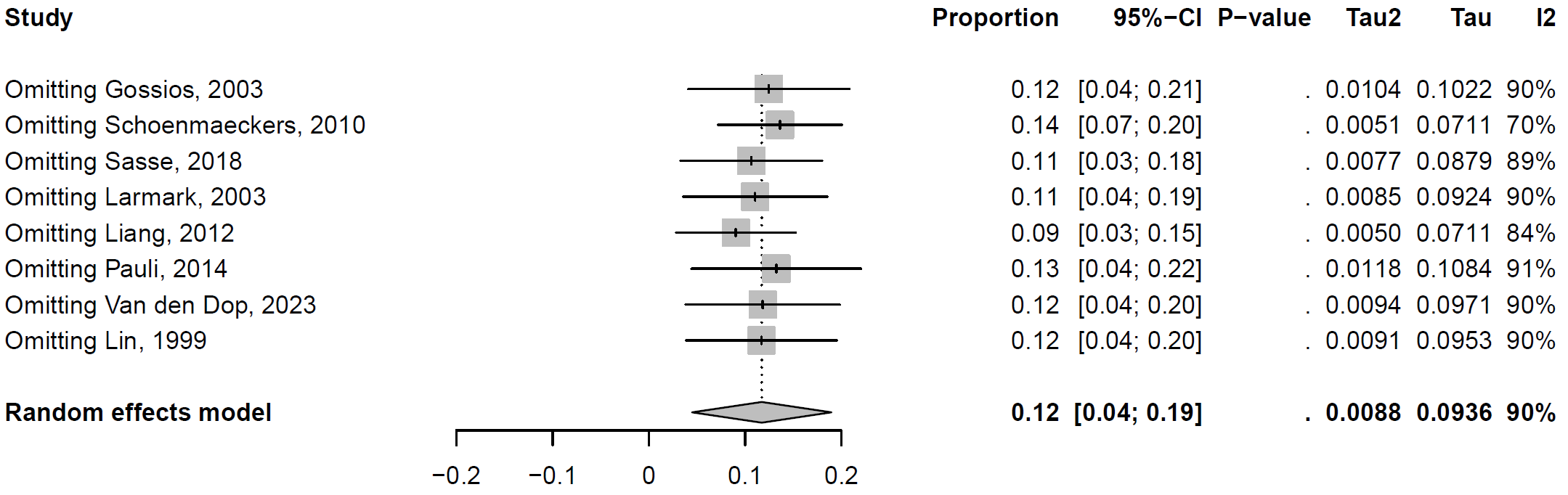


Supplementary Figure 3c) Leave−One−Out Sensitivity Analysis (Random−Effects) for CT Recurrence Rates


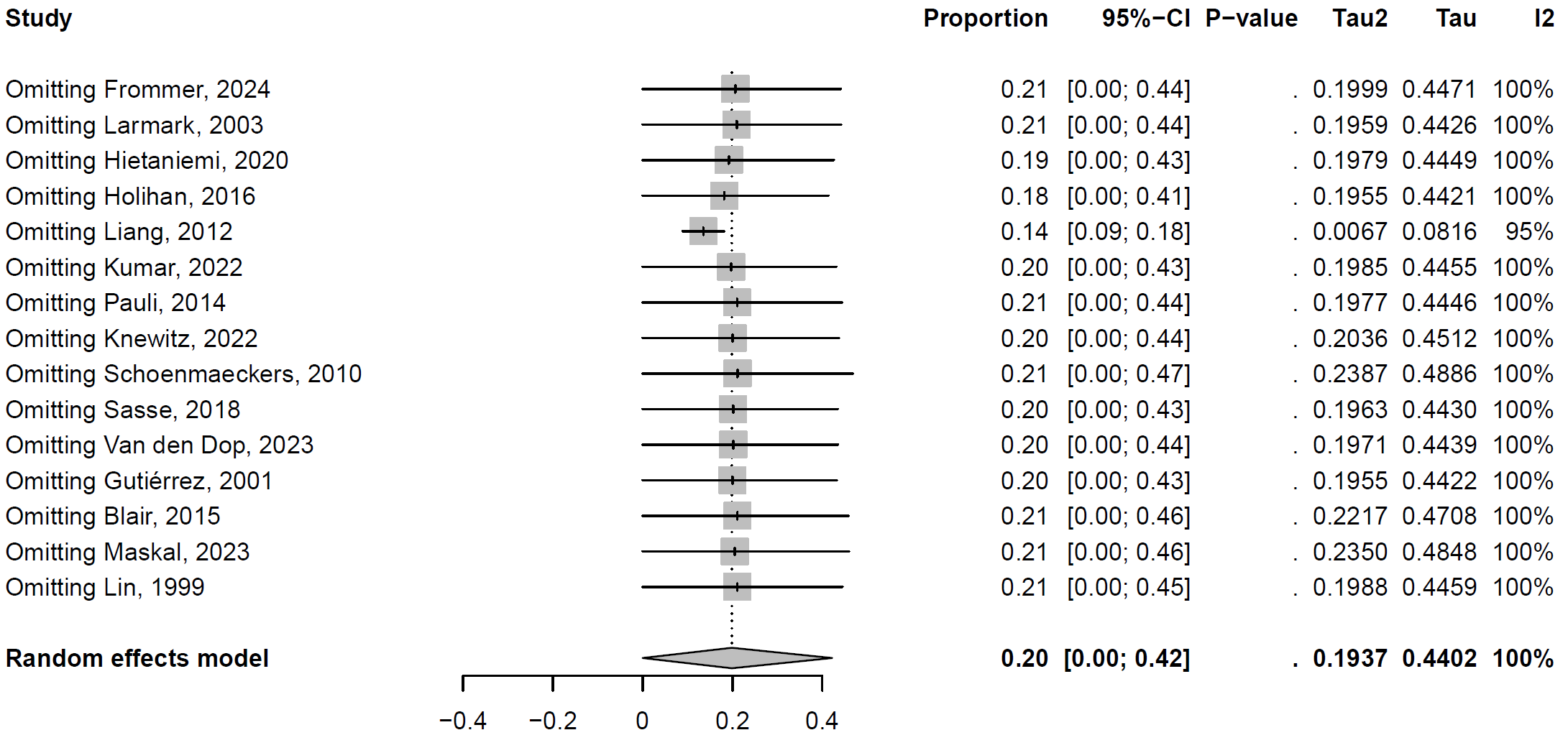


Supplementary Figure 3d) Leave−One−Out Sensitivity Analysis (Random−Effects) for CT Mesh Visualization Rates


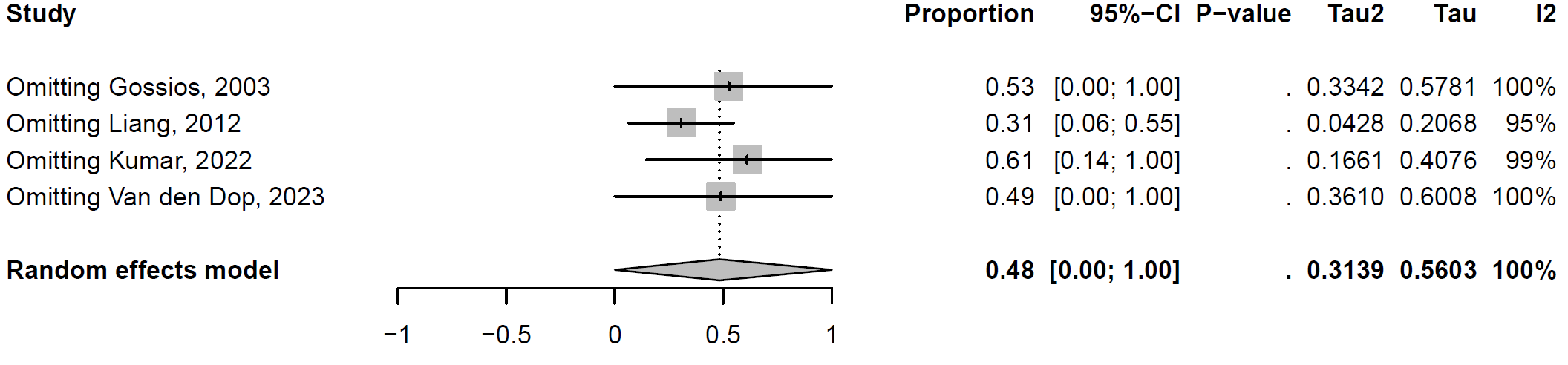


Supplementary Figure 4a) Leave−One−Out Sensitivity Analysis (Random−Effects) for MRI Recurrence Rates


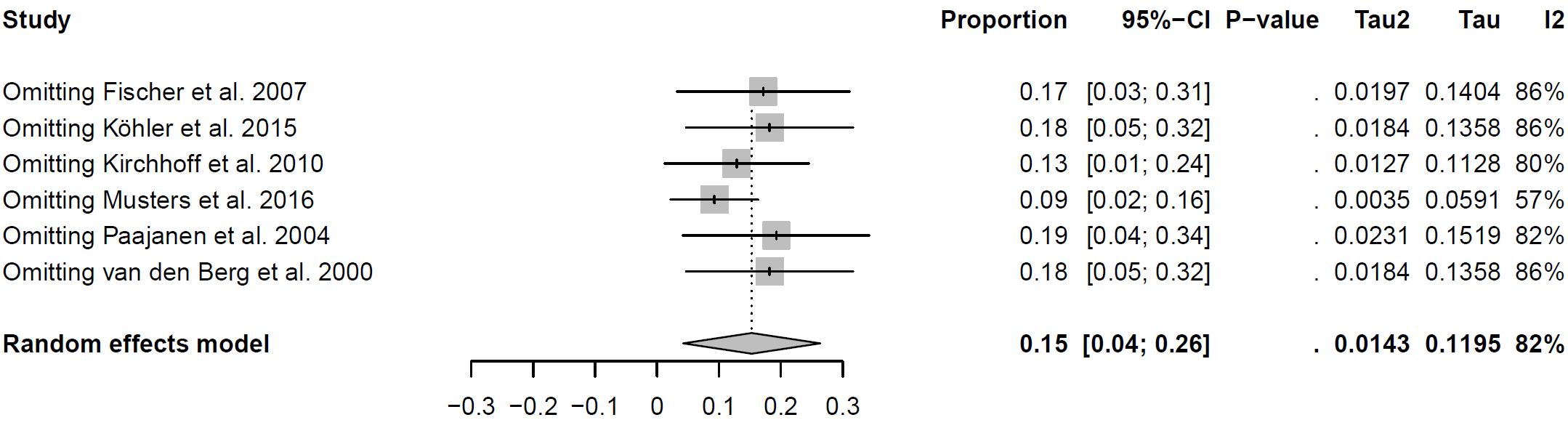


Supplementary Figure 4b) Leave−One−Out Sensitivity Analysis (Random−Effects) for MRI Reoperation Rates


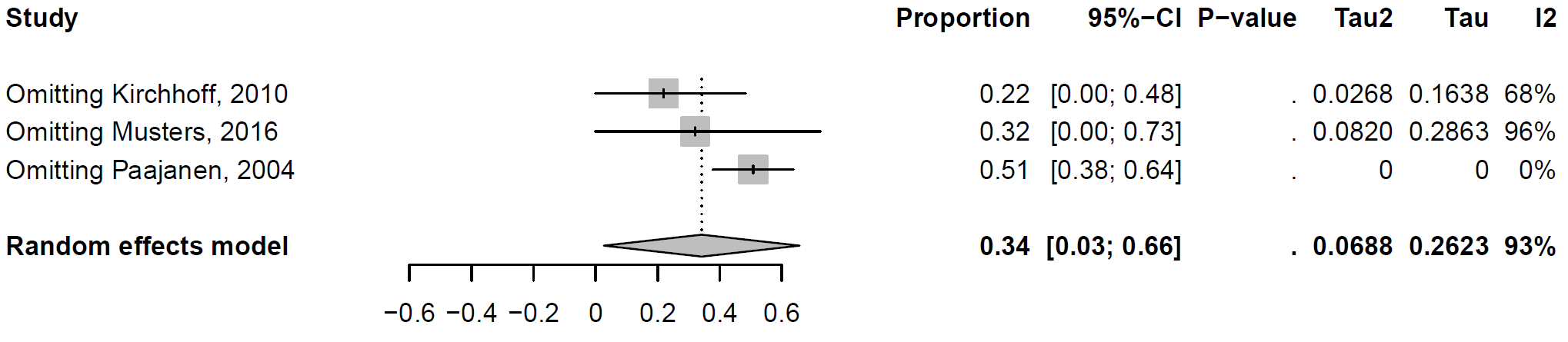


Supplementary Figure 4c) Leave−One−Out Sensitivity Analysis (Random−Effects) for MRI Mesh Visualization Rates


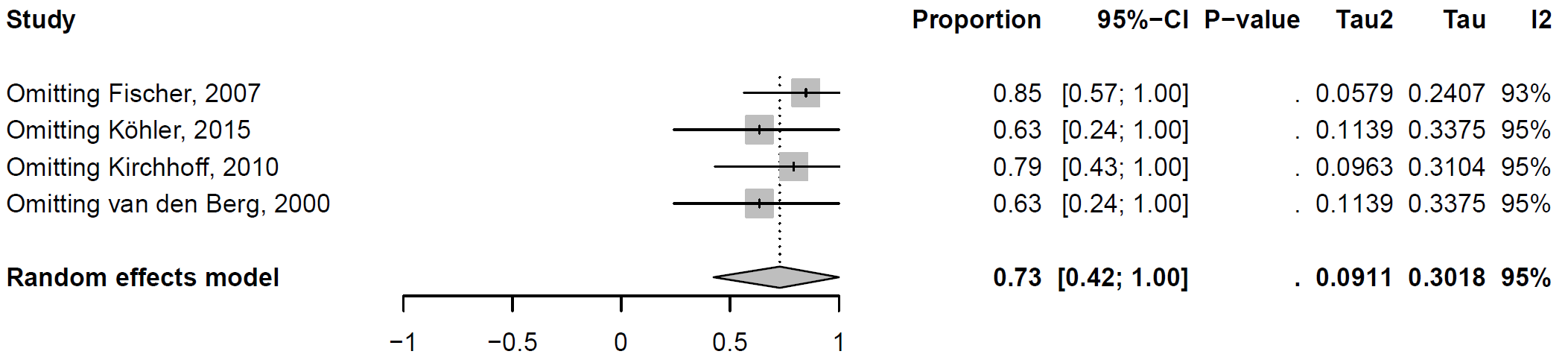


Supplementary Figure 5a) Funnel Plot: Mesh Visualization by Imaging Modality


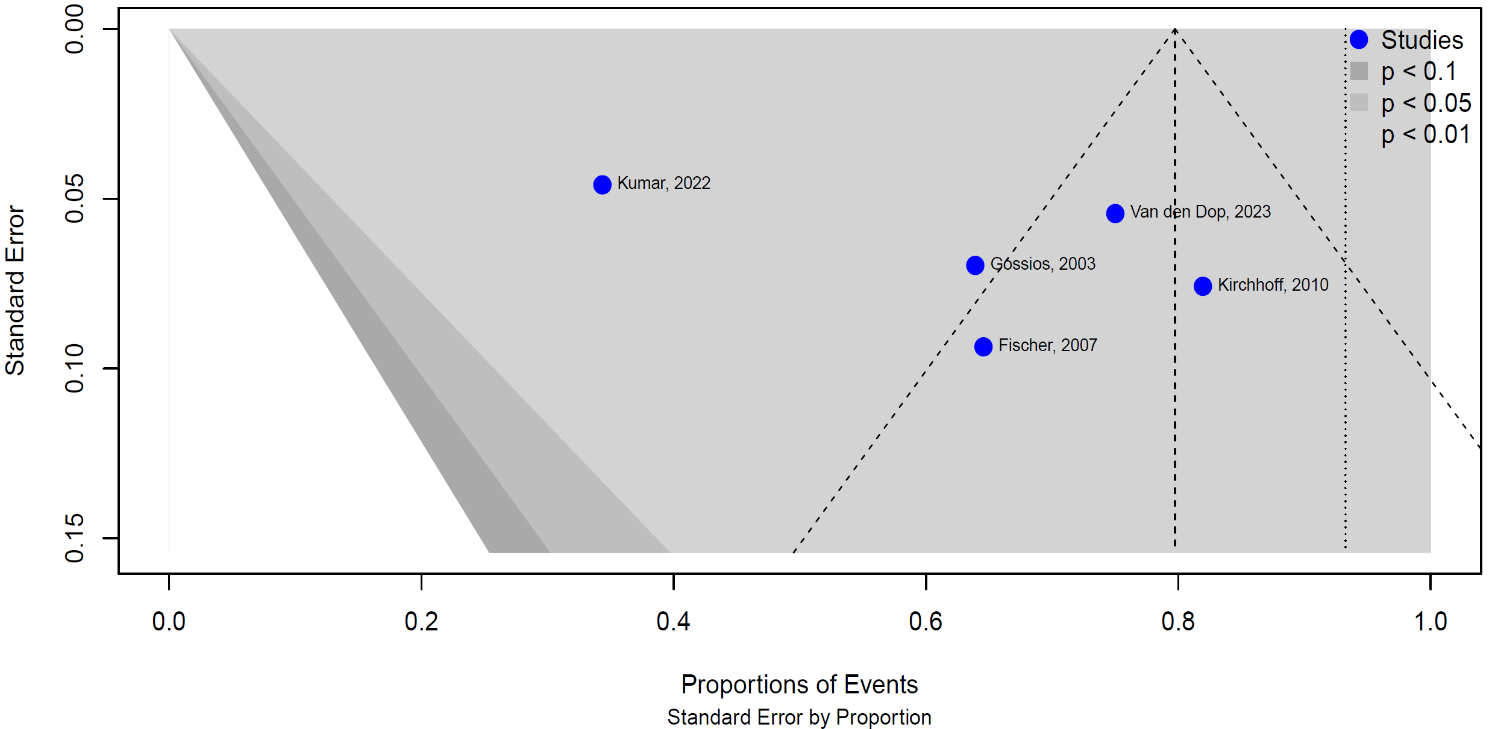


Supplementary Figure 5b) Funnel Plot: Need for Reoperation by Imaging Modality


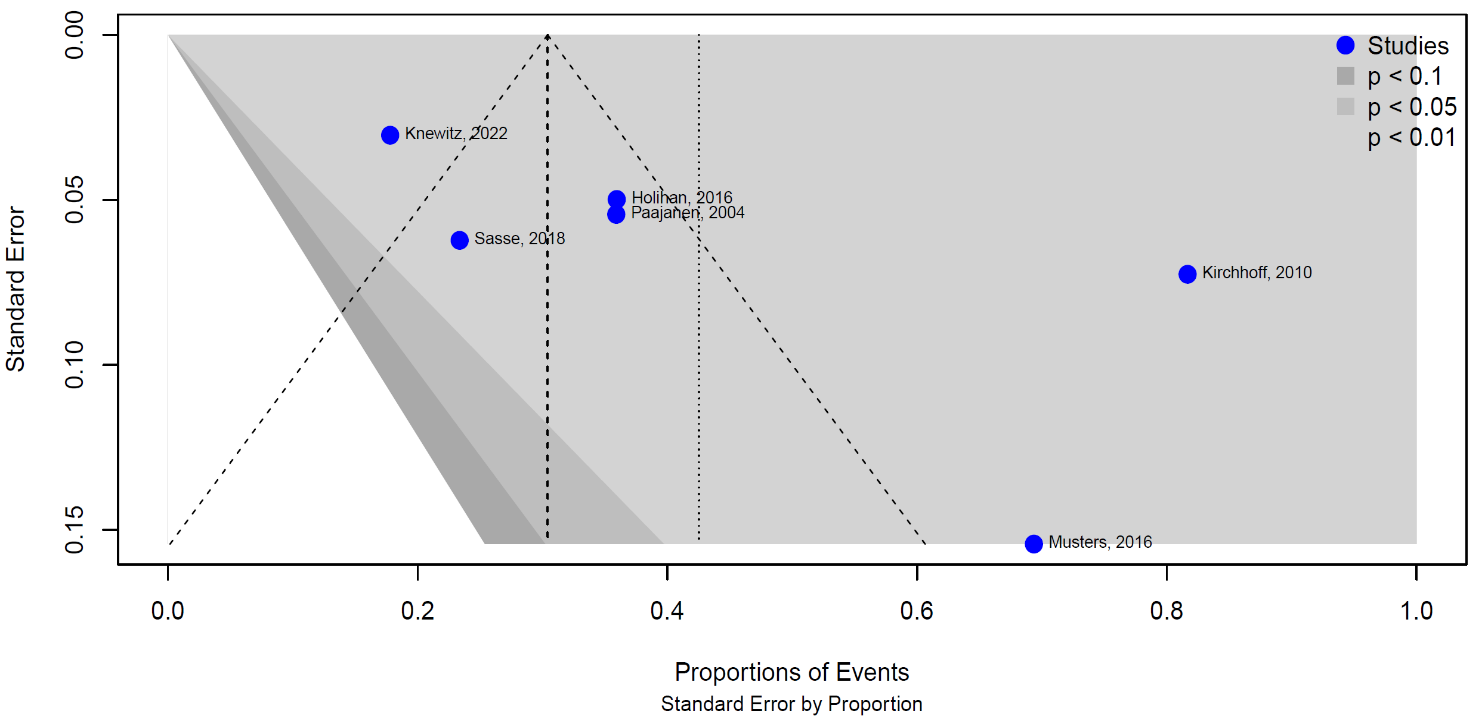


Supplementary Figure 5c) Funnel Plot: Recurrence Across Imaging Modalities


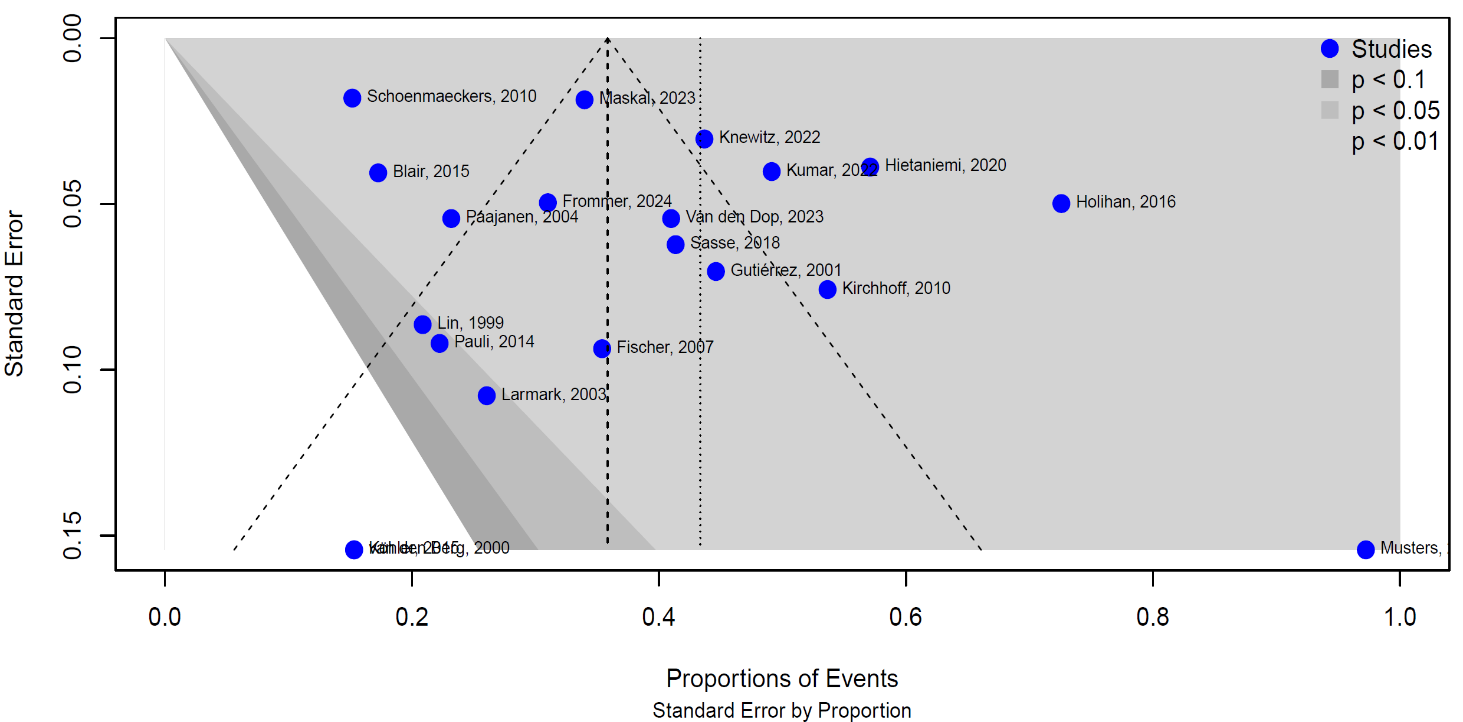


Supplementary Figure 5d) Funnel Plot: Seroma Incidence by Imaging Modality


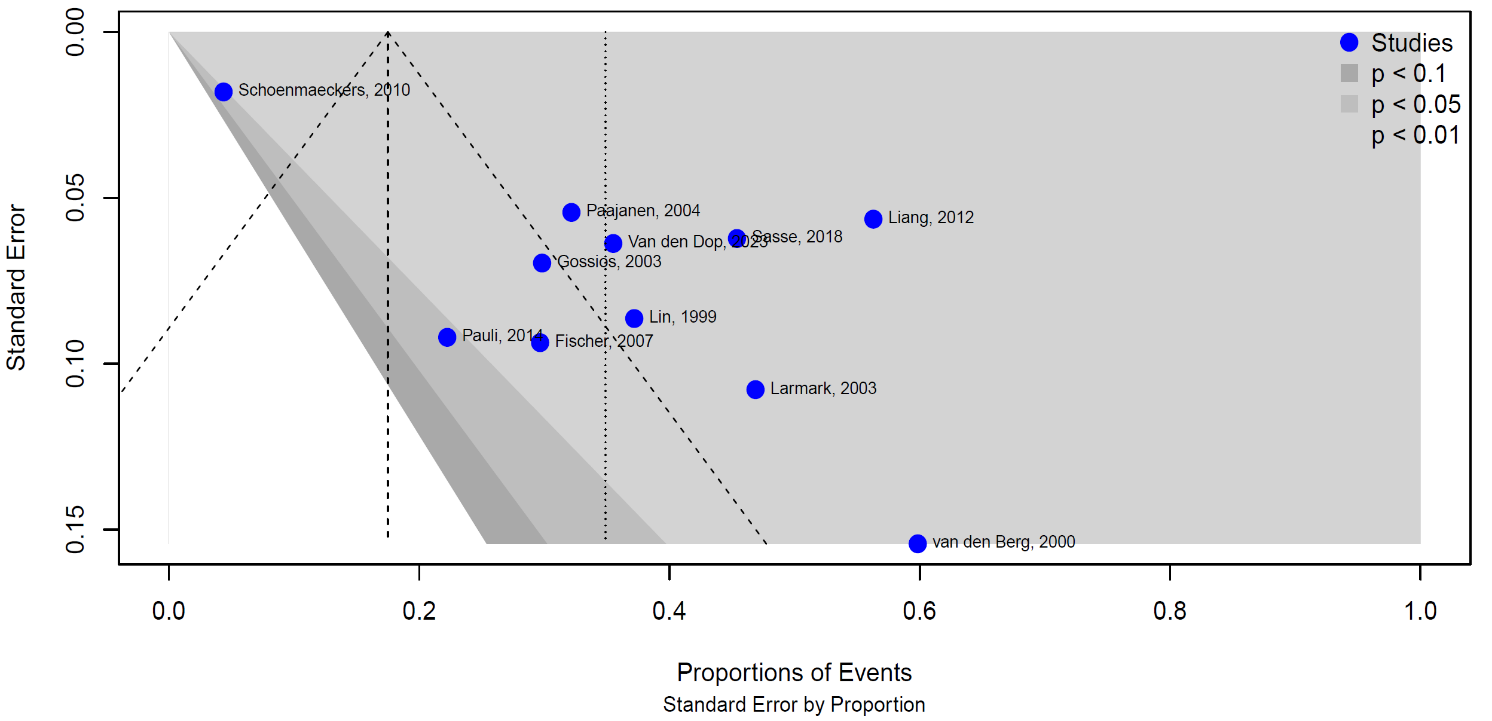

Supplement: Supplementary file 1 — Supplementary file1 (DOCX 2461 KB) [file 10029_2025_3308_MOESM1_ESM.docx]
