# Supplementary material for: Conflict resolution of the beams: CT vs. MRI in recurrent hernia detection: a systematic review and meta-analysis of mesh visualization and other outcomes
Source: Hernia. 2025 Mar 28;29(1):127. doi: 10.1007/s10029-025-03308-9 (PMC11953100; doi:10.1007/s10029-025-03308-9)
Supplement: Supplementary file 2 — Supplementary file2 (DOCX 18 KB) [file 10029_2025_3308_MOESM2_ESM.docx]

| MOOSE Checklist Item | How Addressed in the Study |
| --- | --- |
| Introduction | The background and rationale for the study, as well as the objectives, are clearly described in the Introduction section​. |
| Search Strategy | A comprehensive search strategy across PubMed, Scopus, Embase, and Web of Science is detailed in the Methods section​. |
| Inclusion and Exclusion Criteria | Clear inclusion and exclusion criteria are provided in the Methods section, focusing on studies evaluating CT and MRI in hernia recurrence​. |
| Data Extraction | The process for data extraction, including key variables (recurrence rates, mesh visualization, seroma detection, and reoperation rates), is outlined​. |
| Quality Assessment | Study quality is assessed using the MINORS criteria, as detailed in the Methods section​. |
| Statistical Methods | A random-effects model is employed to pool data, with heterogeneity assessed using I² statistics and subgroup analysis conducted where appropriate​​. |
| Results Presentation | Results are clearly presented with forest plots for each key outcome, including confidence intervals and heterogeneity estimates​. |
| Discussion of Results | The discussion contextualizes findings, considers potential biases, and highlights limitations of the included studies​. |
| Heterogeneity and Sensitivity Analyses | Heterogeneity is assessed, and sensitivity analyses (e.g., leave-one-out analysis) are included to evaluate the robustness of results​. |
| Funding and Conflicts of Interest | The manuscript declares no funding and no competing interests, ensuring transparency​. |

Supplementary Table (1): MOOSE Checklist [23]
