# Supplementary material for: Conflict resolution of the beams: CT vs. MRI in recurrent hernia detection: a systematic review and meta-analysis of mesh visualization and other outcomes
Source: Hernia. 2025 Mar 28;29(1):127. doi: 10.1007/s10029-025-03308-9 (PMC11953100; doi:10.1007/s10029-025-03308-9)
Supplement: Supplementary file 3 — Supplementary file3 (DOCX 15 KB) [file 10029_2025_3308_MOESM3_ESM.docx]

| Database | Search term | Filter | Results |
| --- | --- | --- | --- |
| Embase | ('Computed Tomography':ab,ti OR CT:ab,ti OR 'Computed Tomography Angiography':ab,ti OR 'Magnetic Resonance':ab,ti OR MRI:ab,ti OR Diagnosis:ab,ti OR Diagnose:ab,ti) AND ('Recurrent hernia':ab,ti OR 'hernia recurrence':ab,ti OR 'recurrence of Hernia':ab,ti) | Title/Abstract | 484 |
| SCOPUS | TITLE-ABS ( "Computed Tomography" OR CT OR "Computed Tomography Angiography" OR "Magnetic Resonance" OR MRI OR Diagnosis OR Diagnose ) AND ( "Recurrent hernia" OR "hernia recurrence" OR “recurrence of Hernia” ) | Title/Abstract | 461 |
| WOS | ( "Computed Tomography" OR CT OR "Computed Tomography Angiography" OR "Magnetic Resonance" OR MRI OR Diagnosis OR Diagnose ) AND ( "Recurrent hernia" OR "hernia recurrence" OR "recurrence of Hernia" ) in topic | Topic | 358 |
| PubMed | (((((((Computed Tomography[Title/Abstract]) OR (CT[Title/Abstract])) OR (Computed Tomography Angiography[Title/Abstract])) OR (Magnetic Resonance[Title/Abstract])) OR (MRI[Title/Abstract])) OR (Diagnosis[Title/Abstract])) OR (Diagnose[Title/Abstract])) AND (((Recurrent hernia[Title/Abstract]) OR (hernia recurrence[Title/Abstract])) OR (recurrence of Hernia[Title/Abstract])) | Title/Abstract | 259 |

Supplementary Table (2): MeSH and Emtree terms (Database search)
