# Supplementary material for: Conflict resolution of the beams: CT vs. MRI in recurrent hernia detection: a systematic review and meta-analysis of mesh visualization and other outcomes
Source: Hernia. 2025 Mar 28;29(1):127. doi: 10.1007/s10029-025-03308-9 (PMC11953100; doi:10.1007/s10029-025-03308-9)
Supplement: Supplementary file 4 — Supplementary file4 (DOCX 14 KB) [file 10029_2025_3308_MOESM4_ESM.docx]

| **MRI Studies** | Imaging Modality (CT/MRI) | **Study type** |
| --- | --- | --- |
| Fischer, 2007 [24] | MRI | Cross-sectional |
| Köhler, 2015 [25] | MRI | Prospective Cohort |
| Kirchhoff, 2010 [26] | MRI | Prospective Cohort |
| Musters, 2016 [27] | MRI | Retrospective Cohort |
| Paajanen, 2004 [28] | MRI | Prospective Cohort |
| Özveri, 2021 [29] | MRI | Cross-sectional |
| Hansen, 2015 [30] | MRI | Prospective Cohort |
| van den Berg, 2000 [31] | MRI | Prospective Cohort |

Supplementary Table (3) Types of studies included

| **CT Studies** | Imaging Modality (CT/MRI) | **Study type** |
| --- | --- | --- |
| Frommer, 2024 [32] | CT | Retrospective Cohort |
| Gossios, 2003 [33] | CT | Retrospective Cohort |
| Hietaniemi, 2020 [34] | CT | Retrospective Cohort |
| Holihan, 2016 [35] | CT | Cross-sectional |
| Larmark, 2003 [36] | CT | Prospective Cohort |
| Liang, 2012 [37] | CT | Retrospective Cohort |
| Kumar, 2022 [38] | CT | Case Control |
| Pauli, 2014 [39] | CT | Retrospective Cohort |
| Knewitz, 2022 [40] | CT | Retrospective Cohort |
| Schoenmaeckers, 2010 [41] | CT | Retrospective Cohort |
| Sasse, 2018 [42] | CT | Retrospective Cohort |
| Shemyatovsky, 2020 [43] | CT | Prospective Cohort |
| Van den Dop, 2023 [44] | CT | Prospective Cohort |
| Beck, 2013 [45] | CT | Prospective Cohort |
| Blair, 2015 [46] | CT | Retrospective Cohort |
| Maskal, 2023 [47] | CT | Retrospective Cohort |
| Lin, 1999 [48] | CT | Retrospective Cohort |
| Gutiérrez, 2001 [49] | CT | Prospective Cohort |
