# Supplementary material for: Conflict resolution of the beams: CT vs. MRI in recurrent hernia detection: a systematic review and meta-analysis of mesh visualization and other outcomes
Source: Hernia. 2025 Mar 28;29(1):127. doi: 10.1007/s10029-025-03308-9 (PMC11953100; doi:10.1007/s10029-025-03308-9)
Supplement: Supplementary file 5 — Supplementary file5 (DOCX 16 KB) [file 10029_2025_3308_MOESM5_ESM.docx]

| Recommended Imaging Modality | Clinical Scenario | Rationale |
| --- | --- | --- |
| CT | Preoperative Planning | Detailed anatomical visualization of the hernia and surrounding structures to guide surgical planning. |
|  | Emergency Evaluation | Widespread availability and rapid imaging make CT the preferred choice for urgent assessments. |
|  | Acute Complications | Rapid acquisition and superior detection of acute conditions like bleeding, obstruction, or abscess. |
| MRI | Assessment of Mesh Visualization | Higher sensitivity and specificity in identifying mesh location, shrinkage, and integrity. |
|  | Chronic Mesh-Related Issues | Superior soft-tissue resolution, allowing for detailed evaluation of mesh deformation, fibrosis, and adhesions. |
|  | Long-Term Follow-Up | Avoidance of ionizing radiation and improved visualization of soft-tissue complications. |

Supplementary Table (4): Recommendations for Imaging Modality Selection Based on Clinical Scenarios
