# Supplementary material for: Conflict resolution of the beams: CT vs. MRI in recurrent hernia detection: a systematic review and meta-analysis of mesh visualization and other outcomes
Source: Hernia. 2025 Mar 28;29(1):127. doi: 10.1007/s10029-025-03308-9 (PMC11953100; doi:10.1007/s10029-025-03308-9)
Supplement: Supplementary file 6 — Supplementary file6 (DOCX 13 KB) [file 10029_2025_3308_MOESM6_ESM.docx]

|  | **MRI Studies** | **CT studies** |
| --- | --- | --- |
| **Recurrence** | - z-value: -0.8060  - p-value: 0.4203  - Limit Estimate (b): -0.7178  - Confidence Interval (CI): (-3.5402, 2.1047)  - Interpretation: No significant evidence of publication bias (p > 0.05). | - z-value: 0.7101  - p-value: 0.4777  - Limit Estimate (b): -2.1655  - Confidence Interval (CI): (-3.4734, -0.8576)  - Interpretation: No significant evidence of publication bias (p > 0.05), though the slope suggests a possible trend. |
| **Mesh visualisation** | - z-value: 2.6449  - p-value: 0.0082 (significant)  - Limit Estimate (b): -1.1558  - Confidence Interval (CI): (-2.4974, 0.1858)  - Interpretation: Significant evidence of publication bias (p < 0.05), indicating potential small-study effects. | - z-value: 2.9880  - p-value: 0.0028 (significant)  - Limit Estimate (b): -2.2682  - Confidence Interval (CI): (-4.1132, -0.4233)  - Interpretation: Strong evidence of publication bias (p < 0.05) with a notable negative slope. |
| **Seroma** | - z-value: 0.2962  - p-value: 0.7671  - Limit Estimate (b): -2.6206  - Confidence Interval (CI): (-7.5088, 2.2675)  - Interpretation: No significant evidence of publication bias (p > 0.05). | - z-value: -5.4059  - p-value: < 0.0001 (highly significant)  - Limit Estimate (b): 0.1863  - Confidence Interval (CI): (-0.5378, 0.9104)  - Interpretation: Strong evidence of publication bias (p < 0.05), though the slope is close to zero. |
| **Need for Reoperation** | - z-value: 0.1463  - p-value: 0.8837  - Limit Estimate (b): -1.1299  - Confidence Interval (CI): (-6.2722, 4.0124)  - Interpretation: No significant evidence of publication bias (p > 0.05). | - z-value: -0.4036  - p-value: 0.6865  - Limit Estimate (b): -1.9844  - Confidence Interval (CI): (-6.1602, 2.1914)  - Interpretation: No significant evidence of publication bias (p > 0.05). |

Supplementary Table (5) Egger's Test for Publication Bias
