# Supplementary material for: Conflict resolution of the beams: CT vs. MRI in recurrent hernia detection: a systematic review and meta-analysis of mesh visualization and other outcomes
Source: Hernia. 2025 Mar 28;29(1):127. doi: 10.1007/s10029-025-03308-9 (PMC11953100; doi:10.1007/s10029-025-03308-9)
Supplement: Supplementary file 7 — Supplementary file7 (DOCX 13 KB) [file 10029_2025_3308_MOESM7_ESM.docx]

| **OUTCOME** | **No. OF STUDIES** | **Study Design** | **Risk of Bias** | **Inconsistency** | **Indirectness** | **Imprecision** | **Other Considerations** | **Number of Patients** | **Effect (95% CI)** | **CERTAINTY** | **IMPORTANCE** |
| --- | --- | --- | --- | --- | --- | --- | --- | --- | --- | --- | --- |
| Recurrence (MRI) | 6 | Observational | Low | High (I² = 82%) | None | None | None | 185 | 15% (4% to 26%) | High | High |
| Recurrence (CT) | 15 | Observational | Low | High (I² = 100%) | None | None | None | 1582 | 20% (0% to 42%) | High | High |
| Mesh Visualization (MRI) | 4 | Observational | Moderate | High (I² = 95%) | None | None | Publication Bias | 91 | 73% (42% to 100%) | Low | High |
| Mesh Visualization (CT) | 4 | Observational | Moderate | High (I² = 100%) | None | None | Publication Bias | 331 | 48% (0% to 100%) | Low | High |
| Seroma (MRI) | 3 | Observational | Low | Low (I² = 11%) | None | None | None | 122 | 10% (4% to 15%) | High | High |
| Seroma (CT) | 8 | Observational | Moderate | High (I² = 90%) | None | None | Publication Bias | 1081 | 12% (4% to 19%) | Low | High |
| Reoperation (MRI) | 3 | Observational | Low | High (I² = 93%) | None | None | None | 141 | 34% (3% to 66%) | High | High |
| Reoperation (CT) | 3 | Observational | Low | Moderate (I² = 72%) | None | None | None | 434 | 6% (1% to 11%) | High | High |

Supplementary Table (6) Grade Assessment [51]
